# Revision of *Diplocirrus* Haase, 1915, including *Bradiella* Rullier, 1965, and *Diversibranchius* Buzhinskaja, 1993 (Polychaeta, Flabelligeridae)

**DOI:** 10.3897/zookeys.106.795

**Published:** 2011-06-15

**Authors:** Sergio I. Salazar-Vallejo, Galina Buzhinskaja

**Affiliations:** 1 Depto. Ecología Acuática, ECOSUR, Unidad Chetumal, México; 2 Zoological Institute, Russian Academy of Sciences, Saint-Petersburg, Russia

**Keywords:** Multiarticulated capillaries, body papillae, soft-bottoms

## Abstract

*Diplocirrus* Haase, 1915, includes flabelligerids having cylindrical to club-shaped bodies, with cirriform papillae, multiarticulate chaetae in both parapodial rami, 8 branchial filaments of two types (thick and rarely lamellate, or cirriform), gonopodial lobes in chaetigers 5 or 6, or multiple gonopores along some anterior chaetigers. *Bradiella* Rullier, 1965, has included only the type species: *Bradiella branchiata* Rullier, 1965, described from Eastern Australia. The original description has been overlooked and it lacked enough details on branchial and chaetal features. *Diversibranchius* Buzhinskaja, 1993, with *Diplocirrus nicolaji* Buzhinskaja, 1994, as the type species, was introduced for a similar species from the Japan Sea. These two monotypic genera share the same morphologic features with *Diplocirrus*, and are herein regarded as its junior synonyms. As herein redefined, *Diplocirrus* includes, besides its type species, *Diplocirrus glaucus* (Malmgren, 1867)from Scandinavia : *Diplocirrus branchiatus* (Rullier, 1965), **comb. n**. from Queensland, Australia, *Diplocirrus capensis* Day, 1961 from South Africa, *Diplocirrus erythroporus* Gallardo, 1968 from Vietnam, *Diplocirrus hirsutus* (Hansen, 1882) from Arctic and subarctic regions, *Diplocirrus incognitus* Darbyshire & Mackie, 2009 from South Africa, *Diplocirrus kudenovi* **sp. n.** from off Western Mexico, *Diplocirrus longisetosus* (von Marenzeller, 1890) restricted to the Bering Sea, *Diplocirrus micans* Fauchald, 1972 from deep water off Oregon and Western Mexico, *Diplocirrus nicolaji* (Buzhinskaja, 1994), **comb. n.** from the Japan Sea, *Diplocirrus normani* (McIntosh, 1908), **comb. n.** from Scandinavia, *Diplocirrus octobranchus* (Hartman, 1965), **comb. n.** from off New England, and *Diplocirrus stopbowitzi* Darbyshire & Mackie, 2009 from the Irish Sea.

## Introduction

The delineation of flabelligerid genera has been problematic since Grube (1877a); especially because the eversible anterior end, carrying the branchiae and palps, is rarely exposed. Branchial and chaetal features were employed to propose most genera, but their delineation was not clear-cut in most instances, especially because the branchiae are rarely everted. Thus, *Diplocirrus* was proposed by Haase (1915:194) following some earlier indications by von Marenzeller (1889:130), and by de Saint-Joseph (1898:366). These two authors have commented on the need to separate the species placed in *Stylarioides* delle Chiaje, 1831 by using the branchial arrangement. The species transferred to *Diplocirrus* have four pairs of cirriform, heteromorphic branchiae: the four distal filaments are shorter and thicker, whereas the proximal two pairs include thinner, longer filaments. The branchial filaments in the distal or posterior row differ from those found on the proximal or anterior row; they are basally prismatic due to the fact that when specimens are alive, they are closely packed making a branchial wall. Handling specimens often causes the branchial filaments to separate from the others, such that their lateral connections are not noticed. Further, although the posterior row filaments are thicker than the proximal row filaments, they are dehiscent. The proximal filaments are cirriform separated as two lateral pairs, but are completely free from each other, such that in living or preserved specimens, they look loose and and are deshiscent as well.

It is noteworthy that *Diplocirrus capensis* Day, 1961was described as having all branchiae of the same size and width, neurochaetae distally falcate, and without a cephalic cage. This combination of characters made Day expand the generic diagnosis with some hesitation (Day 1961:510). The whole body was later illustrated (Day 1967:665, Fig. 32.4e), and the emended diagnosis was confirmed. On the other hand, Hartman (1965:178) described *Ilyphagus octobranchus* and made some comments on its affinity with *Diplocirrus capensis*;later, Day (1973:106–107) repeated her observations and because of their proximity, regarded *Ilyphagus* Chamberlin, 1919 as a junior synonym to *Diplocirrus*. As stated elsewhere (Salazar-Vallejo et al. 2008:204), this synonymy cannot be supported because of, among other things, the striking differences in body shape, cephalic cage development, and type of neurochaetae. Further, Darbyshire and Mackie (2009:96), after studying the type material, have found that it has the *Diplocirrus*, typical branchial pattern, and they compiled a table with the morphological characters for most species in the genus.

Chamberlin (1919b:397) introduced *Saphobranchia* for *Stylarioides longisetosus* von Marenzeller, 1890; however, he overlooked the revision by Haase (1915) who had established *Diplocirrus*, including this species into his generic definition. Thus, *Saphobranchia* is a junior synonym of *Diplocirrus*.

Further, eight genera in the polychaete family Flabelligeridae de Saint-Joseph, 1894 have been regarded as monotypic: *Bradabyssa* Hartman, 1967, *Bradiella* Rullier, 1965, *Coppingeria* Haswell, 1892, *Diversibranchius* Buzhinskaja, 1993, *Flabelliderma* Hartman, 1969, *Pantoithrix* Chamberlin, 1919, *Poeobius* Heath, 1930, and *Therochaetella* Hartman, 1967. The proposal of some of these genera may be explained by the lack of a revisionary work that clarifies the generic delimitations in the family. For example, *Flabelliderma* has been redefined recently and it is no longer a monotypic genus (Salazar-Vallejo 2007). *Coppingeria* has been merged into *Stylarioides* delle Chiaje, 1831, as indicated elsewhere (Salazar-Vallejo, in press), and *Therochaetella* has been regarded as a junior synonym of *Trophoniella* Caullery, 1944 (Salazar-Vallejo, submitted).

On the other hand, *Bradiella* has been only known by its type species, *Bradiella branchiata* Rullier, 1965, which was described from Moreton Bay, Queensland, Australia. Spies (1975) studied some specimens from the type locality, but they were identified as *Diplocirrus* cf. *capensis* Day, 1961. This was an unfortunate decision because *Bradiella branchiata* was overlooked by posterior scientists working in the same area. Specimens from a similar species were found in the Sea of Japan by Buzhinskaja (1994); she documented several interesting morphological features, and concluded they were different enough from *Diplocirrus* cf. *capensis*. Thus, she proposed a new genus: *Diversibranchius*, because of the strikingly different branchial filaments.

With this contribution, we revise *Diplocirrus* and regard *Bradiella* and *Diversibranchius* as junior synonyms based on review of type, topotype, and additional materials. *Diplocirrus* is amended and now contains 13 species that live on soft bottoms in sublittoral depths throughout the world.

## Materials and methods

The relative size of notochaetae and their articulation pattern are based on median chaetigers, about chaetiger 10. As in other contributions in this series, specimens were photographed using available equipments; specimens were often temporarily stained with an over-saturated alcoholic methyl green solution. When available, head are depicted in frontal views once branchiae and palps are removed. Plates were prepared by combining several photographs by hand or by using HeliconFocus. Type and non-type materials belong to the following institutions.

**Museum acronyms**

AMAustralian Museum, Sydney.

BMNHThe Natural History Museum, London.

CASCalifornia Academy of Sciences, San Francisco.

ECOSURColección de Referencia, El Colegio de la Frontera Sur, Chetumal.

IRFAInstitut de Recherche Fondamental et Appliquée, Université Catholique de l’Ouest, Angers, France.

LACM-AHFNatural History Museum of Los Angeles County, Allan Hancock Foundation Polychaete Collection.

MNHNMuseum National d’Histoire Naturelle, Paris.

NTMMuseum and Art Gallery of the Northern Territory, Darwin, Australia.

QMQueensland Museum, South Brisbane, Australia.

SAMSouth Australian Museum, Adelaide (GR: Greg Rouse pers. coll.).

SMFForschungsinstitut und Naturmuseum Senckenberg, Frankfurt.

USNMNational Museum of Natural History, Smithsonian Institution, Washington.

ZIRASZoological Institute, Russian Academy of Sciences, Sankt Peterburg.

ZMUBZoologisk Museum, Univesiteet i Bergen, Bergen.

## Results

### Morphology

**Body shape and color.** The body is clavate, subcylindrical, with the anterior few chaetigers often swollen and longer than other segments. Although most species are pale or alternatively take the sediment pigmentation on their body wall, at least along the first few chaetigers, Scandinavian species have been separated by using their overall pigmentation. Thus, *Diplocirrus hirsutus* has been regarded as reddish, at least along few anterior chaetigers, whereas *Diplocirrus glaucus* and *Diplocirrus normani* are grayish. These differences might be due to the sediment particles, and thus be variable depending on the sediment quality rather than a diagnostic feature.

**Cephalic cage.** The first chaetiger is poorly developed in *Diplocirrus* species. However, the relative size of the cephalic cage as well as the number of chaetae per bundle can be used to separate similar species.

**Sediment cover.** The body of the members of the *Diplocirrus* species is variously covered by sediment particles. Fine sediment particles may be adhered to each papilla, whereas larger particles are often trapped between papillae; they are rarely forming a sediment crust. Becausee body papillae are fragile, brushing off the excess of sediment might also remove the papillae, such that particle removal should be done carefully.

**Body papillae.** The relative shape and size of the body papillae has been used to separate similar species. The papillae can be short, being about 3–5 times longer than wide and giving a velvety appearance, or they can be long, being about 8–10 times longer than wide and giving a hirsute outlook. Further, their relative size in relation to notochaetae has been included in the key and descriptions below as an additional means to separate similar species.

**Prostomium.** The prostomium includes a short lobe carrying two pairs of eyes and a posterior projection, the caruncle, which tapers posteriorly, or is distally expanded. To observe this feature, branchial filaments must be removed and to decide if the caruncle is posteriorly expanded, an exploration throughout its length is needed.

**Branchiae.** Branchial filaments are made in two different types and can be separated in two series in relation to the prostomium. The proximal series is the anterior row and the distal one is the posterior row. The posterior row includes prismatic or cuneiform filaments, whereas the anterior row is made of cirriform filaments. The posterior row includes four filaments laterally fused to each other, forming a branchial wall that was illustrated by Haase (1915:29, 197, Fig. 5). This wall is formed because each branchia has two lateral sockets keeping them together, and making their separation difficult. Once separated, each filament is more or less triangular in cross section, but there are two basic modifications; filaments have ciliary bands in most species, whereas in a few of them, filaments are convoluted, with transverse ridges along their surface. Further, because in the latter species the dorsal side is often projected with a long wing, whereas the ventral side might be widened by the presence of multiple blades or lamellae, each filament has a prismatic or cuneiform appearance. These lamellae might be restricted to the proximal or cirriform branchial filaments, even in those species lacking the complex features seen in some posterior branchial filaments. Because they are variable within species, the shape of the filaments and the presence of ciliary bands are not useful for distinguishing genera. These blades are made by either a single series of convoluted filaments, or by a series of transverse filaments arranged as twin blades, but marginally independent of the following blade. Further, these blades can extend over different regions along the back of each branchial filament. The anterior row includes four filaments too, but they are separated in two lateral pairs. Each filament is cirriform, thinner, usually provided with a series of transverse ciliated ridges and, if provided with filamentous blades, hence lamellate, they are more or less restricted to the basal region. As in other flabelligerids carrying two series of branchial filaments as in *Pherusa*, there are some basal branchial knobs between the posterior branchial filaments. They resemble some short, rounded reinforcements present in sabellids or serpulids, and their relative development, whenever evident, might be useful to separate similar species.

**Chaetae.** All chaetae in *Diplocirrus* are multiarticulated with notochaetae thinner than neurochaetae. The multiarticulated notochaetae provide useful diagnostic features by their relative size, in relation to body width, or by the relative size of articles along the chaetae. Thus, articles are regarded as short if they are wider than long, medium-sized if they are as long as wide, and long if they are longer than wide. This variation in the articulation pattern is also present in neurochaetae and because it is a conservative feature, is often used to separate similar species.

**Gonopodial lobes and gonopores.** Adult members of some *Diplocirrus* species carry two projected lobes in chaetigers 4 or 5 which were regarded as neprhidial papillae since Haase (1915). However, nephridial lobes are restricted to the branchial plate, the projected, segmental lobes have a reproductive role and are consequently regarded as gonopodial. Their position in a given chaetiger, as well as their relative color and shape can be used to separate similar species. Three species lack gonopodial lobes, but have several pairs of ventral, rounded, reddish or dark orange structures of unknown function; pending a histological confirmation, they are herein regarded as gonopodial. These multiple paired structures have been described for *Diplocirrus erytrhoporus* Gallardo, 1968, and *Diplocirrus glaucus orientalis* Gibbs, 1971, which is regarded as a junior synonym for the former. Because some species of *Diplocirrus* lack gonopodial lobes, and because they might be present only during reproduction, their presence or absence could not be employed as a generic diagnostic feature, and the multiple gonopores would be in the same condition.

## Systematics

**Class Polychaeta Grube, 1850**

**Order Flabelligerida Pettibone, 1982**

**Family Flabelligeridae de Saint-Joseph, 1894**

### Diplocirrus

Haase, 1915

http://species-id.net/wiki/Diplocirrus

Diplocirrus Haase 1915:194; Day 1967:664–666; Fauchald 1977:116; Darbyshire and Mackie 2009:93.
Saphobranchia Chamberlin 1919:397; proposed for *Stylarioides longisetosa* vonMarenzeller, 1890.
Bradiella Rullier 1965:188.
Diversibranchius Buzhinskaja 1994:231.

#### Type species.

*Trophonia glauca* Malmgren, 1867, by original designation.

#### Diagnosis.

Body clavate or subcylindrical, often anteriorly swollen. Cephalic cage variably developed. Body papillae abundant, short giving a velvety appearance, or very long, giving a hirsute outlook, sometimes adhering sediment particles. All chaetae multiarticulated capillaries; neurochaetae thicker, sometimes falcate. Branchiae sessile, 4 pairs, distal branchiae thicker, often shorter, proximal branchiae thinner, often longer, sometimes basally lamellate. Gonopodial papillae present in chaetiger 4 or 5, or a series of paired ventrolateral gonopores along some anterior chaetigers.

#### Remarks.

Haase (1915) proposed *Diplocirrus* for those species formerly included in *Stylarioides* having two different sizes of branchiae, and multiarticulated capillaries only. Some species currently included in the genus had been previously described in either *Trophonia* or *Stylarioides*. However, as an independent genus, it differs by having two different sizes of branchiae, and all chaetae are multiarticulated capillaries.

Webster and Benedict (1887:730) proposed *Zorus*, with *Zorus sarsi* as the type and only species. They indicated that it had a body anteriorly swollen, becoming thinner posteriorly, only with capillary chaetae, and stated that branchiae and palps arise from an eversible stalk but gave no details on the size relationship of branchiae. Hartman (1961:122) regarded *Zorus* as a junior synonym of *Piromis* Kinberg, 1867. However, because of the body form and chaetal features, it rather resembles *Diplocirrus*, because *Piromis* has few papillae arranged in longitudinal rows and sometimes bifid neurohooks, which were not found in *Zorus*. Webster (1879:46) had already stated the differences among capillary chaetae and ventral hooks when he described another flabelligerid; so, there is no room for any such confusion. The only illustration provided by Webster and Benedict (1887, Pl. 5, Fig. 67), shows a cross section of a middle segment with very long chaetae, and long papillae. These features resemble *Diplocirrus hirsutus* (Hansen, 1879), which is comm in the Bay of Fundi (Appy et al. 1980:32). However, because there is no type material, the generic definition did not include a size relationship of branchial filaments, and the description and illustration lack critical information, *Zorus sarsi* has been regarded as indeterminable (Salazar-Vallejo et al. 2008).

The record by Langerhans (1881:102–103, Figs. 14a–d) of *Brada inhabilis* apparently belongs to *Diplocirrus*, but the illustrations and characters are not clear enough to assign it to any species. The record of *Diplocirrus longisetosus* by Rullier (1964:1094) off Cameroon belongs in *Pycnoderma* Grube, 1877b, likewise *Diplocirrus erythroporus* Gallardo, 1968, includes *Diplocirrus glaucus orientalis* Gibbs, 1971 (:181, no figures; orange globular papillae below each neuropodium in chaetigers 4–14(16)), and might also include the Indian Ocean record of *Diplocirrus glaucus* by Fauvel (1932:186–187, Fauvel 1953:353, Fig. 184a–d).

As stated above, *Diplocirrus capensis* Day (1961:509, Fig. 9a–f, South Africa), was described as lacking cephalic cage, with branchiae of a single kind, and with distally hooked neurochaetae (against generic diagnosis, *cf* Fauvel, Støp-Bowitz). The same different group might include *Diplocirrus* sp A Milligan (1984). The records of the former, originally described from Southern Africa (Day 1967:666, Figs. 32.4e–j; Fauchald 1972:4120), for North Carolina (Day 1973:105–107), and the Gulf of Mexico (Milligan 1984:47.9–11, Figs. 47.5–6), require confirmation to define if they belong to the same species. As stated above, Darbyshire and Mackie (2009) have clarified the branchial features for *Diplocirrus capensis*, whereas the other records remain unsolved.

These differences prompted Day (1961:510), to propose a misfortunate redefinition of *Diplocirrus*, because the branchial features have been employed to establish it by Haase (1915:26, 194). Especially because the posterior row of branchiae are not just thicker than the anterior row filaments; rather, they tend to be closely packed, with each filament laterally fused forming a branchial wall. Further, the cirriform thinner branchiae are contractile, and if they were observed completely relaxed by Day, he might have had the impression that they were of about the same thickness. Later, Day (1973:106–107) modified the generic definition of *Diplocirrus* concluding that it would also include *Ilyphagus* Chamberlin, 1919. However, as stated above, this second emendation is problematic as well, because of marked differences in neurochaetae, because in the species of *Ilyphagus* neurochaetae are aristate neurospines whose handle is made of fused or anchylosed, short articles, and a hyaline fragile tip, whereas in *Diplocirrus* there are only multiarticulate, often falcate, neurochaetae.

*Bradiella* Rullier (1965:190) was compared with *Diplocirrus* and *Brada* Stimpson, 1854. The branchial features were incompletely described (see below); it was regarded as different from these two genera because of the branchiae, and because it lacks gonopodial lobes. The potential differences between *Bradiella* and *Diplocirrus* would be that in *Bradiella* there are no gonopodial lobes in chaetigers 4–5, but gonopodial lobes have not been recorded in some *Diplocirrus* species at all. Further, the surface of branchial filaments is very complex in *Bradiella*, because it is provided with lamellate complex filaments, in contrast to the cirriform or tapering filaments which might barely have some ciliated bands, but some *Diplocirrus* species have a complex lamellar structure along the branchial filaments bases. Spies (1975) studied specimens from the type locality, Moreton Bay, Queensland, Australia, but overlooking the paper by Rullier (1965), identified them as *Diplocirrus* cf. *capensis* Day, 1961. He noticed that the branchiae include eight filaments, not just two as stated by Rullier, with four cirriform and four lamellate filaments. Spies (1975, Pl. 6, Fig. 18) illustrated a (lateral) dorsal spoon-like branchia provided with a flat lateral lobe, and a series of independent branchial blades. Thus, because there are variations in the presence of gonopodial lobes and in the development of lamellar structures in branchial filaments, the only difference to separate the *Bradiella*-like species would be the presence of paired ventrolateral pores. However, because there is no other major difference in chaetal types, *Diplocirrus* and *Bradiella* are regarded as synonyms.

Because of the rediscovery of these peculiar branchial features, Buzhinskaja (1994) established *Diversibranchius*. However, she overlooked Rullier (1965) as well, and compared his specimens with *Diplocirrus*, stressing its resemblance with *Diplocirrus* cf. *capensis*. She found that branchiae were of two types, cirriform and prismatic, or cuneiform, provided with foliose projections, and illustrated that both have convoluted branchial lamellae giving the impression of a series of independent blades, as was illustrated by Spies (1975). *Bradiella* and *Diversibranchius* Buzhinskaja, 1994 resemble each other by having two different types of branchiae, short to long body papillae, and multiarticulated neurohooks. These two genera are herein regarded as junior synonyms to *Diplocirrus*, such that the type species are redescribed, and transferred and newly combined into *Diplocirrus*.

As herein redefined, *Diplocirrus* includes, besides the type species from Scandinavia, *Diplocirrus branchiatus* (Rullier, 1965) comb. n. from Queensland, Australia, *Diplocirrus capensis* Day, 1961 from South Africa, *Diplocirrus erythroporus* Gallardo, 1968 from Vietnam, *Diplocirrus hirsutus* (Hansen, 1882) from Arctic and subarctic regions, *Diplocirrus incognitus* Darbyshire & Mackie, 2009 from South Africa, *Diplocirrus kudenovi* sp. n. from off Western Mexico, *Diplocirrus longisetosus* (von Marenzeller, 1890) restricted to the Bering Sea, *Diplocirrus micans* Fauchald, 1972 from deep water off Oregon and Western Mexico, *Diplocirrus nicolaji* (Buzhinskaja, 1994) comb. n. from the Japan Sea, *Diplocirrus normani* (McIntosh, 1908) comb. n. reinst., from Scandinavia, *Diplocirrus octobranchus* (Hartman, 1965) from off New England, and *Diplocirrus stopbowitzi* Dabryshire & Mackie, 2009, from the Irish Sea.

Two of these species (*Diplocirrus incognitus* and *Diplocirrus stopbowitzi*), have been recently described and only their diagnosis and illustrations are included. On the other hand, three other currently undescribed species are informally characterized but not all have been included in the key because the quality of the materials; one is from Morocco, another one from off Sri Lanka, and the other from Antarctica. The species can be separated using several morphological features as stated below.

#### Key to species of *Diplocirrus* Haase, 1915

**Table:** 

1	Body papillae abundant	2
–	Body papillae scarce, long, tunic looks bare	*Diplocirrus* sp. n. Sri Lanka
2 (1)	Body papillae short, giving a velvety outlook	3
–	Body papillae long, giving a hirsute outlook	11
3 (2)	Body without sand particles	4
–	Body with sand particles	9
4 (3)	Ventrolateral gonopores present in some anterior chaetigers	5
–	Ventrolateral gonopores absent	7
5 (4)	First chaetiger with long chaetae, about half as long as body width; caruncle posteriorly expanded	*Diplocirrus erythroporus* Gallardo, 1968
–	Anterior end with short chaetae, about 1/3–1/5 as long as body width; caruncle posteriorly tapering	6
6 (5)	Median chaetigers with neurochaetae tapering, 22–25 articles, and tip delicately falcate; cirriform branchiae with basal ¼–1/5 with lamella	*Diplocirrus branchiatus* (Rullier, 1965), comb. n.
–	Median chaetigers with neurochaetae barely tapering, 8–11 articles, and tip markedly falcate; cirriform branchiae with basal 1/3–1/2 with lamella	*Diplocirrus nicolaji* (Buzhinskaja, 1994), comb. n.
7 (4)	Papillae digitate, longer than wide, often swollen basally; median chaetigers with 5–6 notochaetae and 4–5 neurochaetae	*Diplocirrus capensis* Day, 1961
–	Papillae hemispherical, about as long as wide	8
8 (7)	Median chaetigers with 5–6 neurochaetae, smaller than notochaetae, with articles 2.0–2.5 times longer than wide	*Diplocirrus kudenovi* sp. n.
–	Median chaetigers with 2–3 neurochaetae, about as long as notochaetae, with articles 7–8 times longer than wide	*Diplocirrus stopbowitzi* Darbyshire & Mackie, 2009
9 (3)	Anterior chaetigers swollen, much wider than following ones; sediment particles scattered	10
–	Anterior chaetigers barely wider than following ones; sediment grains abundant, forming a thin crust	*Diplocirrus* sp. n. Morocco
10 (9)	Lateral papillae 1/5–1/10 as long as longest notochaetae; median chaetigers notochaetae with basal articles poorly defined	*Diplocirrus glaucus* (Malmgren, 1867)
–	Lateral papillae up to 1/3 as long as longest notochaetae; median chaetigers notochaetae with medium-sized articles basally	*Diplocirrus incognitus* Darbyshire & Mackie, 2009
11 (2)	Body without sand particles	12
–	Body with sand particles; median chaetigers with 7–8 notochaetae per bundle; neurochaetae with long articles distally	15
12 (11)	Median chaetigers with notochaetae as long as body diameter; papillae very long, single; chaetiger 1 with 4–5 notochaetae (body often reddish)	*Diplocirrus hirsutus* (Hansen, 1882)
–	Median chaetigers with notochaetae longer than body diameter	13
13 (12)	Median neurochaetae with distal articles barely longer than wide	14
–	Median neurochaetae with most articles 2–4 times longer than wide; no gonopodial lobes	*Diplocirrus micans* Fauchald, 1972
14 (13)	Gonopodial lobes dark (papillae core and tip blackish); body papillae thick, digitate (body often grayish)	*Diplocirrus normani* (McIntosh, 1908), comb. n., reinst.
–	Gonopodial lobes pale; body papillae thin, filiform (body often pale)	*Diplocirrus longisetosus* (von Marenzeller, 1890)
15 (11)	Sand particles restricted to the bases of papillae; neurochaetae with anchylosed region about one-fifth of chaetal length	*Diplocirrus octobranchus* (Hartman, 1965)
–	Sand particles fixed along the papillae; neurochaetae with anchylosed region 1/2–1/3 of chaetal length	*Diplocirrus* sp. n. Antarctica

### Diplocirrus glaucus

(Malmgren, 1867)

http://species-id.net/wiki/Diplocirrus_glaucus

Fig. 1

Trophonia glauca Malmgren 1867:192, Pl. 14, Fig. 78; McIntosh 1915:96–98, Pl. 96, Fig. 2, Pl. 104, Fig. 9 (syn.; simult. Haase 1915; his references stop in 1914).
Diplocirrus glaucus : Haase 1915:195–197, Textfigs. 3–5 (comb. n.); Fauvel 1927:120–121, Figs. 43a–d; Rioja-LoBianco 1931:98–100, Pl. 30; Støp-Bowitz 1948a:25–28, Fig. 6a–c; Hartmann-Schröder 1971:374–376, Fig. 132; Hartmann-Schröder 1996:416–417, Fig. 202; Jirkov and Philippova 2001:358–359, Figs. 1–3; Darbyshire and Mackie 2009:97, Table 1.
Stylarioides flabellata : Fauvel 1946:401 (*non* Sars, 1871).

#### Type material.

**Norway.** Probably lost.

#### Additional material.

**Norway.** One specimen (MNHN-A183), broken in two, without posterior end, anterior end exposed, appendices lost, Solsvick, no further data. Many specimens , Hardangerfjorden (60°10'00"N, 06°00'00"E) separated as follows: 14 anterior fragments (LACM-AHF 2620), Stat. Z20, 7 Jun. 1957, 25–16 m (up to 36 chaetigers, all with multiarticulated neurohooks; in posterior chaetigers with over 10 long articles). Two posteriorly incomplete specimens (LACM-AHF 2622), Stat. Z21, 88–78 m, 7 Jun. 1957. A mature female and a posterior fragment (LACM-AHF 2624), Stat. Z35, 98–104 m, 22 Sep. 1958 (oocytes about 125 µm). Anterior fragment, Stat. Z67, 102 m, 18 Oct. 1958. Seven specimens (LACM-AHF 2683), apparently fixed in alcohol, Stat. Z71, 102–78 m, 20 Oct. 1958 (used for details of branchiae; up to 27 chaetigers, all with multiarticulated neurohooks with articles medium-sized). Two specimens (LACM-AHF 2627), Stat. 121, 66–87 m, 15 Nov. 1958 (used for description). **Faroe Islands.** One specimen (MNHN-A183), anterior fragment, digestive system mostly expulsed from the body, most chaetae broken, RV Pourquoi-Pas? Expedition, off Klaksvik (62°13'26"N, 06°34'43"W), 8–15 m, 30 Jul. 1929. **Sweden.** Many specimens, Tjarno (58.52°N, 11.10°E) and surroundings, Apr. 2002, L. H. Harris, coll., including: One specimen (LACM-AHF 2684) complete, light dark (24 mm long, 2 mm wide, cephalic cage 1,8 mm long, 44 chaetigers; gonad lobes in chaetigers 5 and 6). One anterior fragment (LACM-AHF 2685) with anterior end exposed (used to describe the palp bases and lips). **Russia.** One specimen (ECOSUR), White Sea, 60 m, 28 Jun. 1998, A. Filippova, coll. (7 mm long, 0.8 mm wide, cephalic cage 2 mm long, 21 chaetigers; papillae short, capitate). **Denmark.** Four specimens (USNM-332), damaged, donated by C. Lütken, id. by M. Pettibone (Most chaetae broken; slide with median and posterior chaetiger, median one is only the chaetae. Zero to one chaetae in cephalic cage. Notochaetae very thin, neurochaetae thicker, tips falcate). **Germany.** Two specimens (USNM-175143), North Sea, German Bight, Senckenberg Stat. 24ku, 49.2 m, 12 Aug. 1990, M. Boggemann id.

#### Description.

**Figure 1.**
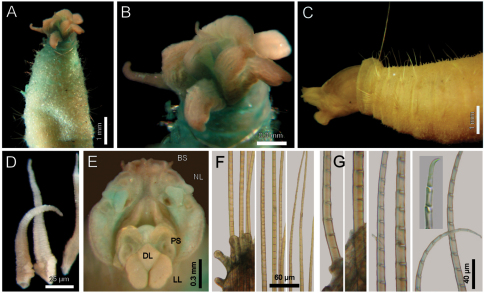
*Diplocirrus glaucus* (Malmgren, 1867). Non-type specimens (LACM-AHF 2683), Norway **A** anterior end, dorsal view, head exposed **B** same, close-up of branchiae showing longitudinal striae **C** another specimen (221), head exposed, anterior end, lateral view **D** same, cirriform branchiae with basal ridges **E** same, head, frontal view, branchiae and pals removed (BS: branchial scars, DL: dorsal lip, LL: lateral lip, NL: nephridial lobes, PS: palp scar), another specimen (LACM-AHF 2627) **F** chaetiger 24, basal, medial and distal notochaetal regions **G** same, basal, medial and distal neurochaetal regions.

Largest specimens (LACM-AHF 2627) pale (some specimens Stat. Z71 with rusty pigmentation in chaetigers 1–3), posteriorly incomplete. Body soft, whitish (Fig. 1A, C), cylindrical, anteriorly swollen, posteriorly tapered; 17–20 mm long, 2–3 mm wide, cephalic cage 2 mm long, 23–27 chaetigers. Tunic with a thin layer of fine sediment grains, papillated. Papillae short, capitate or club-shaped, arranged in 10–12 irregular rows per segment, longer in chaetal lobes, even longer in posterior chaetigers.

Cephalic hood exposed in one specimen (Fig. 1C, LACM-AHF, Stat. 221), almost transparent, smooth. Prostomium low cone (LACM-AHF LH2-514); eyes not seen. Caruncle poorly developed, not reaching the posterior margin of branchial plate, lateral ridges low, median keel not projected (Fig 1E). Palps long, thick; palp keels rounded, reduced. Lateral lips larger, thick, dorsal lip smaller, rhomboid, ventral lip reduced, rounded. Branchiae (LACM-AHF, Stat. Z71) of two different types; posterior branchiae thicker, prismatic, laterally fused to adjacent filaments (Fig. 1B), arranged in a continuous line; anterior branchiae cirriform, slightly longer than posterior branchiae, arranged as two lateral pairs, some with a basal thickening or reinforcement, occupying about 1/6–1/7 of branchial length (Fig. 1D). Palps longer than anterior branchiae. Nephridial lobes, two pairs, placed between posterior and anterior branchiae, each short, rounded (taking methyl-green stain).

Cephalic cage chaetae as long as, or slightly longer than body width. Only notochaetae of chaetiger 1 involved in the cephalic cage, chaetae directed dorsally. Chaetae arranged in a short transverse line; 2–3 notochaetae per ramus. Anterior dorsal margin of first chaetiger papillated, papillae similar to those along the body. Chaetiger 1 short, chaetigers 2–3 longer. Post-cephalic cage chaetigers not elongated, progressively widening to chaetigers 7–8, and then tapering posteriorly. Neurohooks start in chaetiger 1. Gonopodial lobes not seen (other specimens with low, blackish, rounded spots in chaetigers 5–6).

Parapodia reduced, chaetae emerge from the body wall. Parapodia lateral; median neuropodia ventrolateral. Notopodia (Fig. 1F) and neuropodia with slightly elongated papillae in chaetal lobes. Median notochaetae arranged in a short transverse line, as long as about 1/3 body width, 7–8 per bundle; all notochaetae multiarticulated capillaries, articles medium-sized basally, slightly long medially and distally. All neurochaetae multiarticulated hooks with short articles basally, becoming longer medially, tip falcate, smooth (Fig. 1G); median neurochaetae arranged in a transverse line, 4–5 per bundle.

Posterior end tapering, blunt (LACM-AHF-LH-2-522); pygidium with anus dorsoterminal, without anal cirri. A mature female with oocytes, each about 125 µm.

#### Remarks.

*Diplocirrus glaucus* (Malmgren, 1867) is closely allied to *Diplocirrus incognitus* Darbyshire & Mackie, 2009 because both have swollen anterior chaetigers and some sediment particles scattered over the body. They differ in the relative size of lateral papillae and notochaetal articulation; thus, *Diplocirrus glaucus* has smaller papillae (up to one-fifth notochaetal length), and poorly defined basal articles in notochaetae, whereas *Diplocirrus incognitus* has longer papillae (up to one-third notochaetal length) and medium-sized basal articles in notochaetae.

The original description (Malmgren 1867) indicated that the color was variable from bluish-gray to greenish or pale, but the number of chaetae in chaetiger 1 was stated as about 3, which has been used to separate it from similar species. The species was originally described from Bahusiae (Malmgren 1867:192), corresponding with the current Bohuslan (58.88° N, 10.51° E), where the Tjarno Marine Biological Station is, and where some of the specimens used for this description were collected.

#### Distribution.

Northeastern Atlantic Ocean, Russian Northwestern Antarctic seas, in shallow water.

### Diplocirrus branchiatus

(Rullier, 1965) comb. n.

http://species-id.net/wiki/Diplocirrus_branchiatus

Fig. 2

Bradiella branchiata Rullier 1965:188–190, Fig. 7; Darbyshire and Mackie 2009:97, Table 1.
Diplocirrus cf. *capensis*Spies 1975:187, 189, 190, Pl. 3, Fig. 7, Pl. 6, Fig. 18.

#### Type material.

**Australia.** Holotype of *Bradiella branchiata* Rullier, 1965 (AM-W3793), Moreton Bay (27°15'00"S, 153°15'00"E), Brisbane, Queensland, 1.2 km SW of M3 red beacon, coll. Party, 10 Nov. 1961. Two permanent slides (IRFA-W40, -W40’); W40 has three chaetal lobes and a small piece of skin; W40’ has a branchial blade.

#### Additional material.

**Australia.** One anterior fragment (NTM-18913), anterior end exposed, appendages lost, Stat. A16a (12°11.7'S, 136°41.3'E), Melville Bay, 2.7 m, 7 Jul. 1991, Marine Ecology Unit, coll. (14 mm long, 2 mm wide, chaetiger 1 chaetae 0.5 mm long, 17 chaetigers, gonopores in chaetigers 3–12). One complete specimen (QM-G10334), Southwest Rocks, 0.8 km south of Peel Island (27.3° S, 153.21° E), Moreton Bay, Queensland, 6.4 m, shell, grit and sand, Sep. 1970, W. Stephenson, coll. (id. R. B. Spies; dorsally dissected, some parapodia removed, damaged, 13.5 mm long, 3 mm wide, chaetiger 1 chaetae 1 mm long, 21 chaetigers, gonopores pale, in chaetigers 3–8). Anterior fragment (QM-G10379), 1.6 km SE off Southwest Rocks, Peel Island (27.3° S, 153.21° E), Moreton Bay, Queensland, 4–7 m, mud, Mar. 1970, S. Cook, coll. (id. R. B. Spies; dorsally dissected, some parapodia removed, damaged, 38 mm long, 4 mm wide, chaetiger 1 chaetae 1.2 mm long, 18 chaetigers, gonopores reddish, in chaetigers 3–16). Anterior fragment (SAM-GR-201), under Edithburgh Jetty (35°05.172’ S, 137°44.825’ E), Victoria, South Australia, 5 m, in sediment, 1 Mar. 2004, G. Rouse, coll. (it is 12 mm long, 2.5 mm wide, chaetiger 1 chaetae 0.5 mm long, 15 chaetigers, gonopores pale, in chaetigers 3–6).

#### Description.

**Figure 2.**
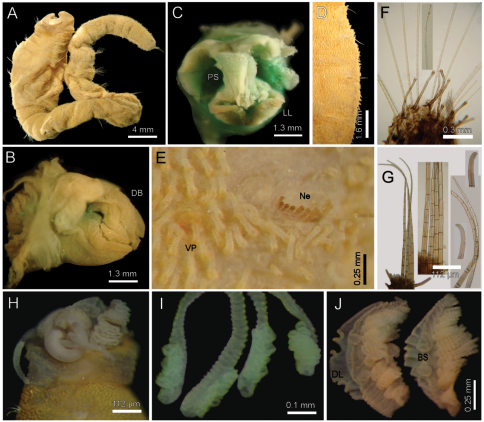
*Diplocirrus branchiatus* (Rullier, 1965), comb. n. Holotype (AM-W3793) **A** entire, ventral view **B** same, head, lateral view (DB: dorsal branchiae) **C** same, frontal view (LL: lateral lip, PS: palp scar) **D** same, right anterior chaetigers 3–6, ventral view **E** same, chaetiger 5 showing broken neurochaetae (Ne) and ventral pore (VP) **F** same, median chaetiger, notopodium (insert: notochaetal distal region) **G** same, median chaetiger, neurochaeta (inserts: anterior neurochaetal tips) **H** non-type specimen (SAM-GR-201), anterior end, head exposed, lateral view **I** same, proximal, cirriform branchiae showing basal ridges **J** same, distal, complex branchiae (BS: branchial sockets, DL: dorsal lamella).

Holotype brown yellowish (other specimens pale, dirty orange or rusty). Body cylindrical, tapering posteriorly (Fig. 2A), contorted, with a previous dorsal longitudinal dissection, and other smaller ones to remove chaetigers 5 and 18; 53 mm long, 6 mm wide, cephalic cage 1.3 mm long, 37 chaetigers. Tunic with abundant papillae, long, cirriform, slightly capitate, with a thin layer of fine sediment particles, forming a thick base, arranged in over 20 irregular bands per segment.

Cephalic hood exposed, with smaller sparse papillae, as long as the following 3–4 chaetigers (swollen in holotype, annulated in QM-10334); cephalic hood margin smooth. Anterior end not everted, observed through the already done dissection. Prostomium elevated, eyes and caruncle not seen because it is bent and covered by the lateral lips (Fig. 2B, C, in SAM-GR201 prostomium flat lobe, no eyes). Palps lost (in SAM-GR201 palps thick, as long as branchiae); palp lobes reduced (thick, rounded in SAM-GR201, and two lateral well-developed lobes. Caruncle projected dorsally to the base of branchiae, lateral ridges elevated, posteriorly separated, laterally expanded. Dorsal lip projected anteriorly; lateral lips thicker; ventral lip reduced. Nephridial lobes in branchial plate not seen).

Holotype with branchial plate damaged. Posterior branchiae compressed, lateral filaments lost, median filament bent towards the mouth, lamellate; cirriform branchiae lost, two lateral scars per side, placed below a dorsal crest. Slide IRFA-W40’ shows a branchial blade made of fused branchial filaments. Another specimen (SAM-GR201), with head slightly exposed (Fig. 2H), branchiae complete of two different types. Posterior row with four prismatic, thicker, lamellate branchiae, tips bare (Fig. 2J); lateral branchiae smaller (one in regeneration), each with dorsal keel reduced, with longitudinal bands, dorsal surface laterally expanded with a thin axis, provided with two rounded lateral lobes; median branchiae larger, dorsal keel large, foliose, markedly corrugated. Distal branchiae with ventral side with a blade made of fused branchial filaments, convoluted, looking like a series of successive blades, but actually made by a single convoluted blade. Anterior branchial row with four thin, cirriform filaments, shorter than palps, arranged in two lateral pairs, each filament with a convoluted lamella along its basal third (Fig. 2I), and successive ciliary bands medial- and distally. Branchial basal lobes between median and lateral branchiae (dorsal), and outside the lateral ones (lateral); dorsal lobes small, rounded, lateral lobes rounded, larger).

Cephalic cage chaetae slightly longer than following ones. First chaetiger displaced dorsally, with multiarticulated capillaries. Notochaetae in a short transverse tuft, with 6–7 multiarticulated capillaries. Anterior dorsal margin of first chaetiger papillated, as following segments. Anterior chaetigers without longer papillae, chaetiger 1 shorter than following ones, chaetal lobes lateral, very close to each other. First 10 chaetigers without marked segmentation between them; following chaetigers shorter, better defined. Ventral gonopores in chaetigers 3–12, orange-red, low papillae (Fig. 2D, E).

Parapodia poorly developed; chaetae emerge from the body wall. Notopodia and neuropodia with papillae as long as other papillae. Noto- and neuropodia close to each other. Median neuropodia lateral, very close to notopodia.

Chaetal transition from first chaetiger to body chaetae abrupt; notochaetae of chaetigers 2–3 large multiarticulated hooks, distal article hooked, entire. All other notopodia with multiarticulated capillaries. Median notochaetae arranged in a longitudinal line. Notochaetae of chaetigers 1 and beyond the third, multiarticulated capillaries; by chaetiger 11, as long as half body width, 10–11 per bundle (6–7 in smaller specimen), each with long articles throughout the chaeta (Fig. 2F). Neurochaetae multiarticulated hooks from chaetiger 1, arranged in a short J-shaped pattern, 4–5 per bundle, each with long articles of about the same length, tips falcate (Fig. 2G), with a hood-like membrane.

Posterior end invaginated in holotype; other specimens with truncated rounded lobe; notochaetae directed posteriorly; without anal cirri.

#### Variation.

Pigmentation varies from pale orange to dark yellowish, or to dirty pink with gonopodial pores reddish or pale. Further, there are two main variations related to body size: papillae are longer in larger specimens, and gonopores become more pigmented, and are probably present along more segments as body enlarges.

#### Remarks.

*Diplocirrus branchiatus* (Rullier, 1965), comb. n. is very similar to *Diplocirrus nicolaji* (Buzhinskaja, 1994), comb. n. because both species have bodies without sediment particles, ventrolateral gonopores along several anterior chaetigers, short chaetae in the first chaetiger, and their caruncle tapers posteriorly. They differ in the relative development of neurochaetae and of the extent of the lamellate area in their cirriform branchiae; thus, in *Diplocirrus branchiatus* median chaetigers have neurochaetae with about 23 articles, tapering to a delicately falcate tip, and the lamellate region might be up to one-fifth of the branchial length, whereas in *Diplocirrus nicolaji*, neurochaetae are barely tapering, having about 10 articles, their tips are markedly falcate, and the lamellate region might extend up to one-third of branchial length.

*Diplocirrus branchiatus* (Rullier, 1965) has been known only through the original description. Spies (1975) studied some specimens from the type locality (herein re-examined); they fit the original description but the anterior end was previously removed. Rullier’s description is fairly complete, though the presence of multiarticulated hooks in notopodia 2–3 was overlooked, as well as the presence of the gonopores. The anterior end has a symmetrical pattern and the original description does not provide complete details about branchiae; however, the drawings show that there were two larger lamellate branchiae (his figure 7C), and that there were smaller lateral branchiae (his figure 7D), but there are no details on cirriform branchiae; they might have been lost during dissection. As originally shown by Rullier (1965), and confirmed by the observation of one permanent slide, branchial blades include a series of parallel filaments; however, they are not arranged as successive, independent blades but rather as a continuous, convoluted, branchial blade. So far, this special type of branchial pattern is only known for a few species in *Diplocirrus*. Further, Rullier illustrated that neurohooks are distally tapering (his figure 7G), but he described them as (p. 190) “plus courtes et recourbées à leur extrémité” (shorter and distally curved), which is the correct description. Spies (1975) made some observations and his drawings are slightly inaccurate in several features: the caruncle does not taper posteriorly, and does not reach the posterior margin of the branchial plate, interbranchial lobes were not illustrated, and the lateral palp lobes were not seen.

#### Distribution.

Originally described from Eastern Australia, *Diplocirrus branchiatus* is present from Northeastern Australia to Southern Australia, in shallow water sediments. The data in the same publication by Rullier (1965), indicate that the type specimen was found in muddy bottoms in shallow depths.

### Diplocirrus capensis

Day, 1961

http://species-id.net/wiki/Diplocirrus_capensis

Diplocirrus capensis Day 1961:509, Fig. 9a–f; Day 1967:666, Figs. 32.4e–j; Day 1973:105–107; Milligan 1984:47.9–11, Figs. 47.5–6; Darbyshire and Mackie 2009:96–98, Table 1 (redescr.).

#### Type material.

The specimens are housed in the South African Museum, Cape Town, but were not made available. Reexamined by Darbyshire and Mackie (2009).

#### Additional material.

**Madagascar.** One specimen (SMF-15355), anterior fragment, damaged, Stat. 11 bis, 47 m, 3 Apr. 1970, R. Plante, coll. (6 mm long, 1 mm wide, cephalic cage chaetae 0.3 mm, 15 chaetigers; gonopores in chaetigers 5–12). Two fragments (SMF-15374), Nosy Iranja, Stat. 4, Benne, 17 Sep. 1966, R. Plante, coll. **Northwestern Atlantic Ocean.** 18 specimens (USNM-51039), damaged, 12 anterior previously dissected or with some parapodia previously removed, and 6 median fragments, off North Carolina, BST 51X (34°20'N, 75°55'W), 165 m, sandy mud, J.H. Day, coll. (larger anterior fragments 6.0–10.5 mm long, 1 mm wide, cephalic cage 0.8–1.0 mm long, 16–28 chaetigers; gonopores not seen).

#### Description.

(modified from Day 1961, 1967 and combined with data from Darbyshire and Mackie 2009. Data from North Carolina specimens in parenthesis, if they differ): Body muddy brown (golden), anteriorly swollen with segmental lines indistinct, tapering posteriorly with better defined segments. Holotype an anterior fragment, 12 mm long, 2 mm wide, no cephalic cage, 18 chaetigers. Tunic papillated; each papillae short, 8-shaped to long, clavate, basally swollen (lateral papillae longer, cirriform).

Cephalic hood not exposed. Prostomium with four small, black eyes. Palps thick, as long as branchiae. Caruncle projected dorsally, not reaching the posterior margin of branchial plate. Lips corrugated, fused. Nephridial lobes in branchial plate not seen. Branchiae very dark, of two types. Posterior row with four wedge-shaped filaments; anterior row branchiae cirriform, separated in two lateral pairs by the caruncle. Interbranchial lobes not seen. Lamellate region difficult to evaluate.

Chaetigers 1–2 with 2–3 fine notochaetae and 4–6 shorter multiarticulated neurochaetae. Anterior dorsal margin of first chaetiger papillated, as following segments; no other modification. Anterior chaetigers without longer papillae, chaetiger 1 shorter than following ones, chaetal lobes lateral, very close to each other. First 10 chaetigers without marked segmentation; posterior segments better defined. Gonopodial lobes not seen.

Parapodia poorly developed; chaetae emerge from the body wall. Notopodia and neuropodia with papillae longer than other body ones. Noto- and neuropodia close to each other. Median neuropodia lateral, very close to notopodia.

Median notochaetae arranged in a longitudinal line, as long as body width, 10–12 (4–6) per bundle, each with short rings basally, long medially and distally. Neurochaetae multiarticulated hooks from chaetiger 1, arranged in a short J-pattern, 6–8 (3–4) per bundle, each with articles of about the same length, tip falcate.

Posterior end unknown.

#### Remarks.

*Diplocirrus capensis* Day, 1961 is closely related to *Diplocirrus kudenovi* sp. n. and *Diplocirrus stopbowitzi* Darbyshire & Mackie, 2009 because their bodies do not incorporate sand particles, and by lacking ventrolateral gonopores. However, these two latter species are provided with hemispherical papillae whereas in *Diplocirrus capensis* papillae are elongate, often basally swollen, but never hemispherical.

The records of *Diplocirrus capensis* by Day (1973:105–107), and Milligan (1984:47.9–11, Figs. 47.5–6) differ from the typical South African form because they have different body color, cephalic cage, larger lateral papillae, and by the relative numbers of chaetae. They might represent a different species but their description as new species must wait for better specimens. There is a similar, apparently undescribed species in the Mediterranean Sea, which has been recorded as *Diplocirrus glaucus* by Fauvel (1937:34, *non* Malmgren, 1867). The materials are damaged (MNHN-406), many chaetae broken, anterior regions smashed or without exposed head, and were collected off Alexandria, Egypt. Better specimens would help clarifiy its affinities with *Diplocirrus capensis*.

#### Distribution.

The distribution for the nominal form is apparently restricted to the Cape province, South Africa, in 11 m; it is questionably recorded from North Carolina, 165 m depth.

### Diplocirrus erythroporus

Gallardo, 1968

http://species-id.net/wiki/Diplocirrus_erythroporus

Fig. 3

Diplocirrus erythroporus Gallardo 1968:108, Pl. 49, Figs. 7–10; Darbyshire and Mackie 2009:97, Table 1.
Diplocirrus glaucus : Fauvel 1932:186–187; Fauvel 1953:353, Fig. 184a–d (*non* Haase, 1915).
Diplocirrus glaucus orientalis Gibbs 1971:181, no figs.

#### Type material.

**Viet Nam.** Holotype (LACM-AHF 306), off Hon Mot Island (12°10'34"N, 109°16'11"E), R.V. Mao Tien, Naga Expedition Stat. 113, 22 m, 10 Feb. 1960.

#### Additional material.

**Viet Nam.** Two specimens (LACM-AHF 2606), Western side of Hon Lon Island (12°12'49"N, 109°14'22"E), R.V. Mao Tien, Naga Expedition Stat. 323, 14 m, 4 Apr. 1960.**Australia.** Two specimens (NTM-18920), one complete, the other without anterior end, Stat. DW69A (12°32.28'S, 130°46.66'E), Darwin Harbor, Australia, 3 m, 17 Mar. 1994, Marine Ecology Unit, coll. (complete: 34 mm long, 3 mm wide, cephalic cage 1.8 mm long, 64 chaetigers, gonopores in chaetigers 4–14). **Yellow Sea.** Anterior fragment (ZISP-10854), plus few chaetigers, Yellow Sea, R.V. Venus, no station data, Chzhan coll.; B. Wu id. as *Brada longicirrata* sp. n. It was 11 mm long, 2 mm wide, cephalic cage 1.5 mm long, 23 chaetigers; four large erect papillae on chaetigers 1–2, one per ramus (resembling a cirrus on each chaetal bundle and hence the name); dorsal ones rise behind the first chaetiger notochaetae whereas the ventral ones stem halfway between the neurochaetae of chaetigers 1 and 2; nephridial pores without pigmentation, in chaetigers 4–12.

#### Description.

**Figure 3.**
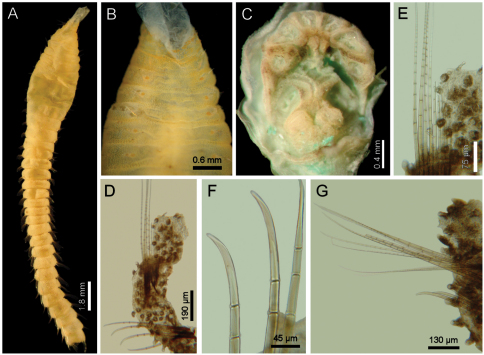
*Diplocirrus erythrophorus* Gallardo, 1968. Holotype (LACM-AHF 11144) **A** dorsal view **B** same, anterior end, ventral view, showing the ventrolateral pores **C** non-type specimen (LACM-AHF 11147), head, frontal view, palps and branchiae removed **D** same, left parapodium, chaetiger 16 **E** same, close-up of notochaetae **F** same, neurochaetal tips **G** same, chaetiger 21, right parapodium.

Holotype an anterior fragment, soft, pale, with dispersed dark brown spots (Fig. 3A). Body cylindrical, anteriorly swollen, posteriorly tapered; 19 mm long, 2.8 mm wide (by chaetiger 7), cephalic cage 0.9 mm long, 33 chaetigers. Tunic papillated, with fine sediment particles.

Cephalic hood exposed, paler than following segments, almost transparent, with smaller papillae; anterior margin papillated, papillae sparse (anterior end dissected in another specimen, LACM-AHF 2606). Prostomium low cone (Fig. 3C); eyes not seen. Caruncle not seen. Palps pale; palp keels reduced. Branchiae of two types, distal row with filaments thick, cirriform; proximal branchiae in two lateral groups, filaments cirriform, thinner, with a thin distal part. Branchiae shorter than palps. Nephridial lobes rounded, low, brownish.

Cephalic cage chaetae as long as 1/3 body width. Only chaetiger 1 involved in the cephalic cage, slightly displaced dorsally. Chaetae arranged in a short lateral line; 3–4 chaetae per ramus. Anterior dorsal margin of first chaetiger papillated, papillae similar to those along the body but with one pair of stiff, long notopodial papillae; posterior chaetigers without long papillae but slightly longer papillae restricted to chaetal lobes.

Chaetigers 1–3 of about the same length (NTM-18920 with chaetiger 2 very thin, chaetiger 3 much longer, almost without papillae). Post-cephalic cage chaetigers not elongated, but progressively widening reaching the widest dimension by chaetiger 7, and then posteriorly reduced. Chaetal transition from cephalic cage to body chaetae gradual; neurohooks start by chaetiger 10. No gonopodial lobes; orange-reddish, disk-shaped gonopores in chaetigers 4–12 (Fig. 3B); in larger specimens along chaetigers 4–14.

Parapodia reduced, chaetae emerge from the body wall (Fig. 3D, G). Parapodia lateral; median neuropodia ventrolateral. Notopodia and neuropodia with slightly longer papillae in chaetal lobes. Median notochaetae arranged in a tuft, oblique to body axis. Median notochaetae as long as ¼ body width, about 9 per bundle; all notochaetae multiarticulated capillaries, articles very short basally, longer medially, becoming medium-sized distally (Fig. 3E). Neurochaetae multiarticulated capillaries resembling notochaetae in chaetigers 1–9; from chaetiger 10, neurochaetae thicker, multiarticulated hooks with short articles basally, becoming long medially, distal article longest, falcate, smooth (Fig. 3F). Median neurochaetae arranged in a transverse line, 4–5 per bundle.

Posterior end missing in holotype; non-type specimen (NTM-18920) with posterior end tapering to a blunt cone; pygidium with anus terminal, no anal cirri.

#### Remarks.

*Diplocirrus erythroporus* Gallardo, 1968 resembles *Diplocirrus branchiatus* (Rullier, 1965), comb. n. and *Diplocirrus nicolaji* (Buzhinskaja, 1994), comb. n. because they all have ventrolateral gonopores along some anterior chaetigers. However, these two latter species have very short chaetae in their first chaetiger, whereas *Diplocirrus erythroporus* has long chaetae. Additionaly, the caruncle of *Diplocirrus erythroporus* is posteriorly expanded unlike that of *Diplocirrus branchiatus* and *Diplocirrus nicolaji*.

The original description (Gallardo 1968) was brief. It indicated that there were six tentacles (branchiae), four larger and two smaller ones, and there were no details on the extent of the cephalic cage. Thus, a redescription was required in order to separate this species from other similar ones in the Indo-Pacific regions. The two additional specimens were one maculated with rounded dark brown spots (11 mm long, 2.5 mm wide, cephalic cage 0.9 mm long, 22 chaetigers, gonopores in chaetigers 4–13), which was dissected to study the anterior end, and another without dark spots (14 mm long, 2.8 mm wide, cephalic cage 1.0 mm long, 23 chaetigers, nephridial pores in chaetigers 4–12; it is a mature female). *Diplocirrus glaucus orientalis* Gibbs, 1971 was described without illustrations; it has orange globular papillae below each neuropodium in chaetigers 4–14(16). This could include the record of *Diplocirrus glaucus* by Fauvel (1932:186–187, Fauvel 1953:353, Fig. 184a–d). It is being regarded as a junior synonym of *Diplocirrus erytrhoporus*.

#### Distribution.

Vietnam, Solomon Islands, Northeastern Australia, in shallow depths (up to 24 m depth).

### Diplocirrus hirsutus

(Hansen, 1878)

http://species-id.net/wiki/Diplocirrus_hirsutus

Fig. 4

**Figure 4.**
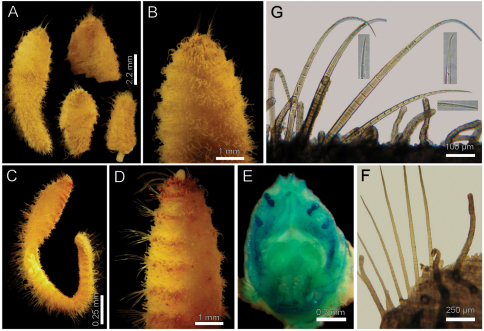
*Diplocirrus hirsutus* (Hansen, 1878). Syntypes (ZMUB-2287) **A** anterior fragments, dorsal view **B** larger syntype, anterior end, dorsal view **C** complete, non-type specimen (ZMUB-27459) **D** same, anterior end, oblique ventral view **E** same, head, frontal view, branchiae and palps removed **F** another syntype, chaetiger 8, right notopodial chaetae **G** same, neurochaetae (inserts: neurochaetal tips).

**Figure 5.**
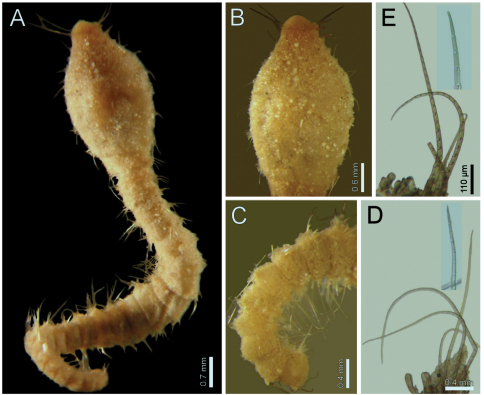
*Diplocirrus incognitus* Darbyshire & Mackie, 2009. Holotype (BMNH 1961.19.694) **A** dorsal view **B** same, anterior end, dorsal view **C** posterior end, dorsal view **D** median chaetiger, notopodium (insert: notochaetal tip) **E** same, neurochaetae (insert: neurochaetal tip).

**Figure 6.**
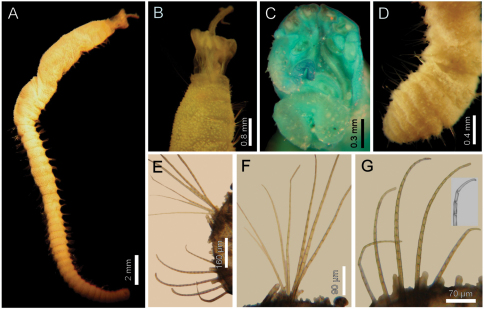
*Diplocirrus kudenovi* sp. n. Holotype (LACM-AHF 2594) **A** lateral view **B** same, anterior end, lateral view **C** same, head, frontal view **D** another specimen, posterior end, dorsal view **E** same, chaetiger 26, right parapodium **F** same, notochaetae **G** same, neurochaetae (insert: neurochaetal tip).

**Figure 7.**
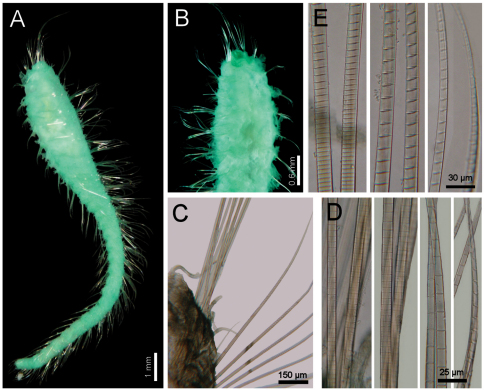
*Diplocirrus longisetosus* (von Marenzeller, 1890) restricted. Neotype (CAS-27933) **A** dorsal view **B** same, anterior end, ventral view **C** paraneotype, chaetiger 25, right parapodium **D** same, basal, medial and distal notochaetal regions **E** same, basal, medial and distal neurochaetal regions.

**Figure 8.**
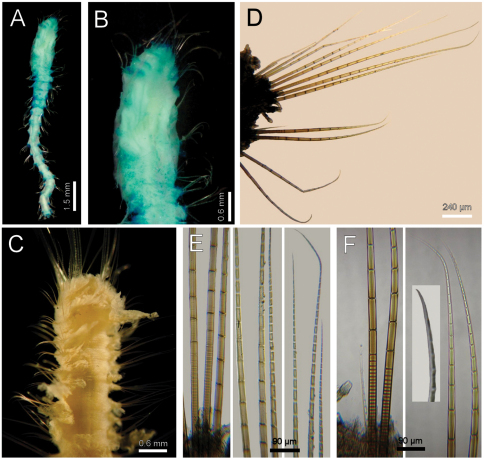
*Diplocirrus micans* Fauchald, 1972. Holotype (LACM-AHF-993) **A** ventral view **B** same, anterior end, ventral view **C** non-type specimen (LACM-AHF-13754), anterior end, dorsal view **D** another non-type specimen (LACM-AHF-13755), chaetiger 14, right parapodium **E** same, basal, medial and distal notochaetal regions **F** same, basal and distal neurochaetal regions.

Trophonia hirsuta Hansen 1878:9–10, Pl. 7, Figs. 1–4; Hansen 1882:38, Pl. 7, Figs. 5–8.
Stylarioides hirsutus :von Marenzeller 1889:129–130 (comb. n.); Ditlevsen 1911:426, Pl. 29, Fig. 11, Pl. 31, Figs. 23, 24.
Diplocirrus hirsutus : Haase 1915:198–200 (comb. n.); Støp-Bowitz 1948a:28–30, Fig. 7, Støp-Bowitz 1948b:37–38, map; Wesenberg-Lund 1950:35; Fauchald 1972:412; Jirkov and Philippova 2001:359, Figs. 1–7; Darbyshire and Mackie 2009:97, Table 1.

#### Type material.

**Norway.** Syntypes (ZMUB-2287), four anterior fragments and a dissected anterior end, two previously dissected, NMH Expedition, Stat. 18 (62°44'N, 01°48'E), and Stat. 31 (63°10'N, 05°00'E) (syntypes yellowish, incomplete, 4–5 notochaetae in chaetiger 1; 8–9 notochaetae in median chaetigers; 10 transversal rows of papillae in chaetiger 10; gonopodial lobes not visible).

#### Additional material.

**Norway.** Two specimens (ZMUB-25216), Norkse Nordhavs. Expedition, Stat. 262 (no data) (two anterior fragments, dried out). Five specimens (ZMUB-27459), NMH (N. Nordhosk Expedition, Stat. 326 (no data), Hansen, coll. (complete 19–22 mm long, 2.5–2.7 mm wide, cephalic cage 2.0–2.5 mm long, 29–37 chaetigers; gonopodial lobes in chaetigers 5–6 in two specimens).

#### Description.

Larger syntype pale, soft, yellowish (Fig. 4A, B). Body club-shaped, swollen anteriorly, progressively narrowing to chaetiger 12, then cylindrical to the end of the fragment (and body; Fig. 4C)); 10 mm long, 3.5 mm wide, cephalic cage chaetae 2 mm long, 20 chaetigers. Tunic papillated, fine sediment particles on papillae basis only (other specimens with sediment cover towards the tip). Papillae long, abundant, capitate, with basal sediment making a rounded lobe (Fig 4B), about 10 transverse rows in chaetiger 10, much longer dorsally, longest about 2/3 as long as notochaetae.

Anterior end observed in a previously dissected specimen and in non-type specimen. Cephalic hood short, smooth, margin smooth. Prostomium low cone, grayish, eyes barely pigmented (Fig. 4E), difficult to be seen in syntype or non-types. Caruncle not observed in syntype, weakly defined in non-types. Palps thick, longer than the only available cirriform branchia; palp bases rounded, projected. Lateral lips projected, thick, well-developed, dorsal and ventral lips reduced. Branchiae mostly lost, scars remain; posterior row with thicker scars, anterior row with a single cirriform branchiae without basal blades (all cirriform, posterior ones slightly thicker, smooth). Nephridial lobes rounded, elevated, separating anterior and posterior branchial rows (taking methyl green stain deeply).

Cephalic cage chaetae shorter than body width. Chaetiger 1 involved in the cephalic cage, chaetae arranged in short dorsolateral lines, with 4–5 noto- and 9–10 neurochaetae per bundle. Anterior dorsal margin of first chaetiger papillated; anterior chaetigers with papillae longer than those present in following chaetigers. Chaetigers 1–3 progressively longer. No chaetal transition from cephalic cage chaetae to body chaetae; all neurochaetae multiarticulate falcigers but first chaetigers with shorter articles. Gonopodial lobes not seen in syntypes (oval, bare, pale areas in chaetigers 5–6 in non-types; Fig. 4D).

Parapodia lateral, poorly developed, chaetae emerge from the body wall; median neuropodia ventrolateral. Notopodia without conical lobes. Noto- and neuropodia distant to each other.

Median notochaetae arranged in a longitudinal, transverse, short line; all notochaetae multiarticulated capillaries (Fig. 4F), medium-sized articles basally, longer medial- and distally; 8–9 notochaetae per bundle in median chaetigers (up to 14 in non-types), about as long as body width. All neurochaetae multiarticulated hooks, markedly tapering subdistally (Fig. 4F); basal articles short, ill-defined, longer medial- and distally, but diminishing in size towards the tip, 5–6 per bundle.

Posterior end, observed in non-type specimens, truncate (Fig. 4C); pygidium with anus terminal, without anal cirri.

#### Remarks.

*Diplocirrus hirsutus* (Hansen, 1878) resembles *Diplocirrus longisetosus* (von Marenzeller, 1890) and *Diplocirrus normani* (McIntosh, 1908), comb. n. because they have bodies provided with long papillae but without sand particles. Their main difference lies in the relative length of notochaetae in median chaetigers, because the latter two species have notochaetae markedly longer than body width, whereas in *Diplocirrus hirsutus* they are about as long as body width.

Haase (1915:199) noticed the cinnamon-red color for specimens of this species. The available specimens show a concentration of the pigment towards the anterior end, making a thin crust surrounding papillae and chaetae. Thus, it is not the basic color of the organism but rather some adsorbed minerals on these structures and, whenever this pigmentation is present, chaetae are darker, which indicates that the minerals are either ingested and later used for chaetal formation, or adsorbed to chaetae as well as over the tunic. This pigmentation should rely on the minerals available in the sediments, and therefore should not be used as a diagnostic feature.

#### Distribution.

Originally described from Norway, it ranges in Arctic and Subarctic environments in shallow water. The Antarctic records by Hartmann-Schröder and Rosenfeldt (1989:71–72; 1991:74–75) are questionable.

### Diplocirrus incognitus

Darbyshire & Mackie, 2009

http://species-id.net/wiki/Diplocirrus_incognitus

Fig. 5

Diplocirrus incognitus Darbyshire and Mackie 2009:99–102, Figs. 3B, 4, Table 1.

#### Diagnosis.

Body anteriorly swollen (Fig. 5A). Papillae abundant, short, giving a velvety oultlook, with scattered sediment particles (Fig. 5B, C). Lateral papillae 1/3 as long as longest notochaetae. Median notochaetae with long articles (Fig. 5D). Neurochaetae with long articles, tips barely curved (Fig. 5E).

#### Remarks.

As stated above, *Diplocirrus incognitus* Darbyshire & Mackie, 2009 resembles *Diplocirrus glaucus* (Malmgren, 1867), because both have bodies anteriorly swollen and few sediment particles spread over the body. They differ in the relative size of lateral papillae and on the notochaetal basis articulation; thus, in *Diplocirrus incognitus* papillae are longer (up to one-third notochaetal length), and notochaetal bases have medium-sized articles, whereas in *Diplocirrus glaucus* papillae are smaller (up to one-fifth notochaetal length) and notochaetal bases have poorly-defined articles.

#### Distribution.

South Africa, offshore, in muddy bottoms of about 100 m depth.

### Diplocirrus kudenovi sp. n.

urn:lsid:zoobank.org:act:1EED4521-10A1-4EA5-87AE-FDCCF9E389A7

http://species-id.net/wiki/Diplocirrus_kudenovi

Fig. 6

#### Type material.

**Eastern Pacific Ocean.** Holotype (LACM-AHF 2594) and 14 paratypes (LACM-AHF 2595), Southern Bay, Isla Cedros, Baja California, Mexico, RV Velero IV, Stat. 2026 (20°05'00"N, 115°19'45"W), 16 fathoms, mud and sand, 19 Apr. 1951 (best paratypes: 8.0–22.5 mm long, 1–2 mm wide, cephalic cage 1.0–1.5 mm long, 24–49 chaetigers).

#### Additional material.

**Gulf of California.** One anterior fragment (LACM-AHF 2596), damaged, off southeastern tip of Isla Angel de la Guarda, Baja California, Mexico, Stat. P-71–59 (29°20.0'N, 113°11.2'W), 40 fathoms (7 mm long, 1.5 mm wide, cephalic cage 1.5 mm long, 19 chaetigers).

#### Description.

Holotype (LACM-AHF2594), without posterior end, soft, whitish (Fig. 6A). Body club-shaped, anteriorly swollen, progressively narrowing to chaetiger 15, then cylindrical, tapering to the end of the fragment; 19 mm long, 2 mm wide, cephalic cage 1.5 mm long, 47 chaetigers. Tunic papillated, fine sediment particles on papillae basis only. Papillae short, abundant, capitate, with basal sediment making a rounded lobe, about 13–15 irregular rows in anterior chaetigers (about 10 rows in median chaetigers), slightly longer dorsally and in posterior chaetigers; in median chaetigers papillae as long as 1/5–1/6 notochaetal length.

Anterior end completely exposed, slightly damaged (Fig. 6B). Cephalic hood short, smooth, margin smooth. Prostomium low, pale, eyes not seen. Caruncle poorly developed, lateral ridges low, median keel reduced, not continued to the posterior margin of the branchial plate (Fig. 6C). Palps lost in holotype (pale in one paratype), palp bases rounded. Lateral lips well developed, dorsal lip reduced, ventral lip rounded. Branchiae mostly lost, branchial scars on branchial plate, arranged in two rows, posterior row with 4 thicker branchial scars, anterior row discontinuous, two narrower branchial scars, one long cirriform branchia left. Nephridial lobes rounded, separating posterior and anterior branchiae.

Cephalic cage chaetae shorter than body width. Chaetiger 1 involved in the cephalic cage, slightly displaced dorsally; chaetae arranged in short dorsolateral lines, with 2 noto- and 4 (–6) neurochaetae. Anterior dorsal margin of first chaetiger papillated; anterior chaetigers without especially long papillae. Chaetigers 1–3 progressively larger. No chaetal transition from cephalic cage to body chaetae, all neurochaetae similar, but first chaetiger with shorter articles. Gonopodial lobes present in chaetiger 5 (or 5 and 6 in some paratypes), a transverse papillae-free area.

Parapodia lateral, poorly-developed, chaetae emerge from the body wall (Fig. 6E); median neuropodia ventrolateral. Notopodia without conical chaetal lobes. Noto- and neuropodia distant to each other.

Median notochaetae arranged in a longitudinal, short line; all notochaetae multiarticulated capillaries, short articles basally, long medially and distally (Fig. 6F). About 6–8 chaetae per bundle, 1/2–2/3 as long as body width. All neurochaetae multiarticulated hooks, feebly-defined short articles basally, medial- and distally with long articles, distally falcate (Fig. 6G); neurohooks arranged in a transverse line, with 5–6 per bundle.

Posterior end (observed in a paratype) tapering to a rounded lobe (Fig. 6D); pygidium with anus terminodorsal, without anal cirri.

#### Etymology.

This species is named after Jerry D. Kudenov, who has studied several polychaete families on a world-wide basis, and especially for his series of publications on the polychaetes from the Gulf of California, which have been very useful for many researchers working in the region, including one of us (SISV). The epithet is a noun in the genitive case.

#### Type locality.

Southern Bay, Isla Cedros, Baja California, México, in mud-sand bottoms, at 16 fathoms depth.

#### Remarks.

*Diplocirrus kudenovi* sp. n. is very similar to *Diplocirrus stopbowitzi* Darbyshire & Mackie, 2009, because both have bodies without sand particles, with papillae hemispherical, and by lacking ventrolateral gonopores. They differ in chaetal features, especially regarding neurochaetae; thus, in *Diplocirrus kudenovi*, median chaetigers have 5–6 neurochaetae and each has articles about twice as long as wide, whereas in *Diplocirrus stopbowitzi*, there are 2–3 neurochaetae and each has longer articles, being about seven times longer than wide.

#### Distribution.

Western Mexico, in both sides of the Baja California Peninsula, in subtidal waters.

### Diplocirrus longisetosus

(von Marenzeller, 1890) restricted

http://species-id.net/wiki/Diplocirrus_longisetosus

Fig. 7

Stylarioides longisetosus von Marenzeller 1890:5 Fig. 3, von Marenzeller 1892:426–427.
Diplocirrus longisetosus : Haase 1915:200–202, Textfigs. 6–7 (*partim*); Ushakov 1955:307(1965:285), Fig. 114G, H; Darbyshire and Mackie 2009:97, Table 1.

#### Type material.

**Gulf of Alaska.** Neotype (CAS-27933), and paraneotypes (CAS), off Pitt Point, Alaska, Stat. 1546 (71°19.5'N, 152°58.0'W), 55 m, sandy silt, 11 Aug. 1977, R.E. Ruff, coll. and id. (paraneotypes 10–14 mm long, 1 mm wide, cephalic cage 2.5–3.0 mm long, 25–31 chaetigers).

#### Additional material.

**Bering Sea.** Two anterior fragments (ZIRAS-27133), Providence Bay, Stat. 74 (no specific data), 18 m, mud, P. Uschakov, coll. (10.0/10.5 mm long, 2.0/2.5 mm wide, cephalic cage chaetae 3.0/2.5 mm long, 18/16 chaetigers; gonopodial lobes in chaetiger 5).

#### Description.

Neotype complete (CAS-27933), pale yellowish. Body club-shaped, anteriorly swollen, progressively narrowing to chaetiger 13, then cylindrical, tapering to the posterior end (Fig. 7A); 12 mm long, 1.5 mm wide, cephalic cage 2.5 mm long, 33 chaetigers. Tunic papillated, detached in several portions, with fine sediment particles. Papillae pale, cirriform, sparse, about 5–6 transverse rows in median chaetigers, slightly longer dorsally; in median chaetigers about 1/5 as long as notochaetae.

Anterior end modifications observed in a paraneotype. Cephalic tube short, smooth, margin apparently smooth. Prostomium low, pale, eyes black, small. Caruncle not seen. Palps pale, thick, deeply furrowed, as long as branchiae; palp keels reduced. Lips damaged by dissection. Branchiae thick, cirriform, sessile on branchial plate; posterior branchiae thicker, anterior branchiae cirriform, two thinner filaments per lateral group. Nephridial lobes very thin, long, placed below the posterior row central filaments.

Cephalic cage chaetae 1/5 as long as body length, or 2/3 as long as body width. Chaetigers 1–2 involved in the cephalic cage; chaetae arranged in short, dorsolateral lines, 5(–8) noto- and 5 neurochaetae per bundle. Anterior dorsal margin of first chaetiger papillated; anterior chaetigers without especially long papillae. Chaetigers 1–3 progressively larger. Post-cephalic cage chaetigers not elongated. No chaetal transition from cephalic cage to body chaetae, all neurochaetae similar. Gonopodial lobes present in chaetiger 5, low, round, pale lobes, covered by small papillae, difficult to be seen even after methyl green staining (Fig. 7B).

Parapodia lateral, poorly-developed, chaetae emerge from the body wall (Fig. 7C); median neuropodia ventrolateral. Notopodia 1–2 with low, conical, chaetal lobes directed forward, remaining parapodia without conical lobes. Neuropodia 1–4 with low, conical chaetal lobes. Noto- and neuropodia distant to each other.

Median notochaetae arranged in a transverse horizontal C-shaped pattern; all notochaetae multiarticulated capillaries, short articles basally, medium-sized medially, long distally (Fig. 7D). About 11(–13) chaetae per bundle, twice as long as body width. All neurochaetae multiarticulated capillaries, very short articles basally, well-defined, medium-sized medially, long distally (Fig. 7E); tips straight; arranged in a transverse line, 8–9 per bundle.

Posterior end tapering to a rounded lobe; pygidium with anus terminal, without anal cirri.

#### Neotype locality.

Off Pitt Point, Alaska, 55 m, sandy silt.

#### Remarks.

As currently restricted, *Diplocirrus longisetosus* (von Marenzeller, 1890),closely resembles *Diplocirrus micans* Fauchald, 1972 and *Diplocirrus normani* (McIntosh, 1908), comb. n. These species have notochaetae longer than the body width, and long papillae without sand particles, although *Diplocirrus micans* separates from the other two species by having neurochaetae with long articles, and because it lacks gonopodial lobes. Then, *Diplocirrus longisetosus* and *Diplocirrus normani* differ especially in the relative body color, papillae and gonopodial lobes, and on the relative resolution of neurochaetal basal articles. In *Diplocirrus longisetosus*, papillae and gonopodial lobes are pale, and basal neurochaetal articles are well-defined, whereas in *Diplocirrus normani*, the body is grayish, and papillae and gonopodial lobes are darker or blackish, whereas neurochaetal basal articles are poorly-defined.

Further, *Diplocirrus longisetosus* was described from Providence Bay, Russia, in the Bering Sea, with a single anterior fragment. Haase (1915:200) studied the supposed holotype (which is now lost), an additional specimen sent him by von Marenzeller, probably coming from Spitzbergen, Norway, and an additional broken specimen. This combination resulted in a mixture of morphological features and the species has been recorded from several localities in the Arctic Ocean as well as in the Northern Atlantic and Northern Pacific. Consequently, a redescription and proposal of a neotype is needed to clarify if there is more than one species. Støp-Bowitz (1948:32) noticed the nephridial lobes in the branchial plate, but he regarded them as accessory branchiae.

After the International Code of Zoological Nomenclature (1999, Art. 75), a neotype is being designated because there is no name-bearing type specimen, and because of the confusion between the above two species requires a designation to objectively define *Diplocirrus longisetosus*. Consequently, in order to satisfy the qualifying conditions (Art. 75.3), it must be stated that this designation will clarify the taxonomic status, a description and illustrations have been presented to ensure the recognition of the species. Further, collection managers in several German museums were contacted in order to find the type material for this species, but none exists. On the other hand, the neotype fits the characteristics originally noticed in the species, it was found in a locality with ecological conditions similar to the ones prevailing in the original type locality, and has been deposited in the California Academy of Sciences.

#### Distribution.

Originally described from Providence Bay (64°30'N, 173°30'W), Russia, these specimens come from Northern Alaska, about 1,200 km away, but despite the distance between them, these localities share the same environmental conditions, and the incomplete topotype specimens have most of the same morphological features.

### Diplocirrus micans

Fauchald, 1972

http://species-id.net/wiki/Diplocirrus_micans

Fig. 8

Diplocirrus micans Fauchald 1972:218–219, Pl. 44, Figs. a–e; Darbyshire and Mackie 2009:97, Table 1.

#### Type material.

**Eastern Pacific Ocean.** Holotype (LACM-AHF992), off Natividad Island, Baja California, RV Velero IV, Stat. 7229 (27°54'25"N, 115°40'00"W), 957–942 fathoms, 31 Dec. 1960.

#### Additional material.

**Eastern Pacific Ocean.** Several fragments (LACM-AHF 2615), off Natividad Island, Baja California, RV Velero IV, Stat. 7231 (from 27°24'00"N, 115°12'15"W, to 27°23'17"N, 115°13'45"W), 1355–1312 fathoms, green mud, 1 Jan. 1961. Median fragment (LACM-AHF 2612), off Natividad Island, Baja California, RV Velero IV, Stat. 7249 (27°36'25"N, 115°56'25"W), 2050–2027 fathoms, red clay and rock, 4 Jan. 1961. Two specimens (LACM-AHF 2611), 44 miles, 192 degrees N from Cabo Corrientes Lighthouse, RV Velero IV, Stat. 13754-70 (19°41"15"N, 105°53’00” W), 1220 fathoms, Campbell grab, 18 Jan 1970 (25–30 mm long, 1.2–1.5 mm wide, cephalic cage 1.5–3.0 mm long; chaetiger 1 with 2–3 noto- and 5–6 neurochaetae per bundle, 39 chaetigers; female with oocytes 125 µm). An anterior fragment (LACM-AHF 2611a), 35.3 miles 205 degrees T (T=true north) from Cabo Corrientes Lighthouse, RV Velero IV, Stat. 13755-70 (19°51'30"N, 105°58'00"W), 1400 fathoms, Campbell grab, 18 Jan 1970 ( 7 mm long, 1.5 mm wide, cephalic cage chaetae 7 mm long; chaetiger 1 with 4–5 noto- and 5–6 neurochaetae).

#### Description.

Holotype pale, damaged, without posterior end (in regeneration?), several parapodia removed, many chaetae broken. Body slightly swollen anteriorly, tapering posteriorly (Fig. 8A); 11 mm long, 1 mm wide, cephalic cage 1 mm long, 26 chaetigers. Tunic papillated, with abundant, fine sediment particles adhered. Papillae short, abundant (most eroded), cylindrical, longer in first chaetiger and in chaetal lobes, less than 1/3 chaetal length (very long in LACM-AHF 2615, as long as half notochaetal length).

Anterior end not exposed; not dissected to avoid further damage. Cephalic cage chaetae as long as body width. Chaetigers 1–2 involved in the cephalic cage; chaetae arranged in short, lateral lines, 2 chaetae per ramus. Anterior dorsal margin of first chaetiger papillated. Anterior chaetigers without long papillae. Chaetigers 1–3 progressively larger; notopodia with suprachaetal conical lobes. Post-cephalic cage chaetigers not elongated. Chaetal transition from cephalic cage to body chaetae abrupt; multiarticulared neurochaetae start in chaetiger 3. Gonopodial lobes not seen (Fig. 8B).

Parapodia porly-developed, chaetae emerge from the body wall (Fig. 8D). Parapodia lateral; median neuropodia ventrolateral. Noto- and neuropodia low, rounded lobes, very close to each other. All notochaetae multiarticulated capillaries, articles short basally, become long medially and distally (Fig. 8E). Median notochaetae arranged in a short, transverse line, holotype with 2–3 per bundle (other specimens with 8–9 chaetae per bundle), twice as long as body width. Neurochaetae multiarticulated capillaries in chaetigers 1–2; multiarticulated, thicker neurospines start in chaetiger 3, arranged in a transverse line, 4 per bundle (up to 8 in larger fragments Stat. 7231). Neurochaetae with short articles basally, become long medially, slightly decreasing their length distally; tips slightly falcate (Fig. 8F).

Posterior end unknown.

#### Remarks.

*Diplocirrus micans* Fauchald, 1972 resembles other species with abundant papillae and long chaetae such as *Diplocirrus longisetosus* (von Marenzeller, 1890), and *Diplocirrus normani* (McIntosh, 1908), comb. n. However, *Diplocirrus micans* separates from the two other species because its neurochaetae have long articles, and there are no gonopodial lobes, whereas the two other species have distal articles barely longer than wide, and gonopodial lobes.

The record by Fauchald and Hancock (1981:36) was based on a single, damaged specimen collected off Oregon, United States. The specimen (LACM-AHF 2616) resembles *Diplocirrus micans* but it is brittle, apparently it has dried out in the past, so the conical lobes in first few chaetigers cannot be confirmed. However, this specimen has many more chaetae per bundle, especially in the anterior end, and articles are much longer than in *Diplocirrus micans*, so it may be a different species, but the specimen is in poor shape and more specimens are required to describe it.

#### Distribution.

Western Mexico, in deep water (1900–2800 m depth).

### Diplocirrus nicolaji

(Buzhinskaja, 1994) comb. n.

http://species-id.net/wiki/Diplocirrus_nicolaji

Fig. 9

Diversibranchius nicolaji Buzhinskaya 1994:231, Figs. 2–7; Darbyshire and Mackie 2009:97, Table 1.
Flabelligeridae from Japan: Rouse and Pleijel 2001, Plate 11, Fig. f.

#### Type material.

**Northwestern Sea of Japan.** Holotype (ZIRAS-48504), Vostok Bay (42°30'N, 133°00'E), Peter the Great Bay, Russia, 7 m, muddy sand, 26 Oct. 1989, G. Buzhinskaja, coll. Several paratypes (ZIRAS-48506), five anterior fragments (four with anterior end exposed, variously damaged), and several median fragments, Vostok Bay (42°50'N, 132°45'E), Peter the Great Bay, Russia, 7 m, muddy sand, 26 Oct. 1989, sample 2, G. Buzhinskaja & S. Kiyashko, coll. (anterior fragments 5.5–12.0 mm long, 0.7–2.0 mm wide, 12–24 chaetigers, chaetiger 1 notochaetae 0.3–0.6 mm, 10–22 transversal rows of papillae, gonopodial pores in chaetigers 3–7(–8, 9, 14 one each). Five paratypes (ZIRAS-48507), Vostok Bay (42°50'N, 132°45'E), Peter the Great Bay, 3m, muddy sand, 21 Sep. 1989, G. Buzhinskaja, coll. (6–9 mm long, 0.6–1.0 mm wide, 10–18 chaetigers, chaetiger 1 notochaetae 0.4–0.5 mm, 12–20 transversal rows of papillae, gonopodial pores in chaetigers 3–9(–10 in 2 paratypes, –11 in one; gut sediment particles heterogeneous, up to 0.6 mm long).

#### Additional material.

**Northwestern Pacific Ocean. Northwestern Sea of Japan, Peter the Great Bay, Russia.** One specimen (ZIRAS-2/48505), Vostok Bay (42°50'N, 132°45'E), 7 m, muddy sand, 26 Oct. 1989, G. Buzhinskaja, coll. Five specimens (ZIRAS-3/48506), one beheaded, Vostok Bay (42°50'N, 132°45'E), 7m, muddy sand, 26 Oct.1989, G.Buzhinskaja & S.Kiyashko, coll. Five specimens (ZIRAS-4/48507), Vostok Bay (42°50'N, 132°45'E), 3m, muddy sand, 21 Sept.1989, G.Buzhinskaja, coll. One specimen (ZIRAS-5/48508), beheaded, Posyet Bay (42°30'N, 131°00'E), 3 m, muddy sand, among *Zostera asiatica*, diving, sample from 0.25 m2, 10 Mar. 1966, A.N. Golikov, coll. Three anterior fragments (ZIRAS-6/48509), beheaded, Tikhaya Bay, Posyet Bay (42°30'N, 131°00'E), 3m, muddy sand, among *Patiria pectinifera* and *Chaetopterus*, 3 Mar. 1966, diving, sample from 0.1 m2, A.N. Golikov, coll. One specimen (ZIRAS-7/48510), Tikhaya Bay, Posyet Bay (42°30'N, 131°00'E), 4–5 m, muddy sand, among *Patiria pectinifera* and *Chaetopterus*, 21 Apr. 1965, diving, sample from 0.3 m2, L. Chislenko, coll.

#### Description.

**Figure 9.**
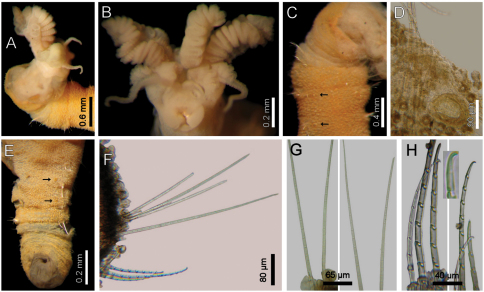
*Diplocirrus nicolaji* (Buzhinskaja, 1994), comb. n. Holotype (ZIRAS-48504) **A** anterior end, oblique lateral view, body in ventral view **B** same, head, frontal view, palps and one posterior branchia removed **C** same, anterior chaetigers, ventral view (arrows indicate ventral pores) **D** paratype (ZIRAS-48506), posterior end, ventral view (arrows indicate ventral pores) **E** same, posterior end **F** same, chaetiger 13, right parapodium **G** Same, basal and distal notochaetal regions **H** same, basal and distal neurochaetal regions (insert: neurochaetal tip).

Holotype (ZISP-48504) orange yellow, slightly macerated, without posterior end. Body anteriorly swollen, posteriorly tapering; 19 mm long, 1.9 mm wide, no cephalic cage (chaetiger 1 notochaetae 0.3 mm), 30 chaetigers. Tunic densely covered by papillae (Fig. 9A, C, E); papillae short, most 8-shaped, others digitate, with fine sediment adhered to their base, about 12 rows per segment.

Cephalic hood exposed, as long as the following 4 chaetigers, with small, sparse papillae, cephalic hood margin smooth. Prostomium low, eyes not seen. Palps thick, slightly longer than branchiae; palp lobes reduced, rounded. Other features from paratypes. Caruncle projected dorsally to the base of posterior branchiae, tapering, lateral lobes elevated, posteriorly fused. Dorsal lip projected, lateral lips thicker, ventral lip reduced. Nephridial lobes in branchial plate not seen (Fig. 9B).

Branchiae of two different types (Fig. 9A, B). Posterior row with four prismatic, thicker, lamellate branchiae, lamella reaching the tips; lateral branchiae of the same size, with dorsal keel rounded, reduced, with longitudinal bands and laterally expanded dorsal surface, with a thin axis, branchial lateral margins with two rounded, sucker-like sockets; median branchiae with dorsal keel as those present in lateral branchiae, not foliose, corrugated. All posterior branchiae with a series of successive transverse blades on their ventral side; in median branchiae, all laterally fused making a single convoluted blade; in lateral branchiae the transverse blades laterally free. Anterior row with four thin, cirriform branchiae, shorter than palps, arranged in two lateral pairs, each filament with a convoluted lamella along its basal third, and successive ciliary bands medial- and distally. Interbranchial lobes small, between median and lateral branchiae (dorsal), and outside the lateral ones (lateral); dorsal lobes small, rounded, lateral lobes rounded, slightly larger).

First chaetiger displaced dorsally, notochaetae slightly longer than following ones. Notochaetae arranged in a short, oblique line with 2 multiarticulated hooks. Anterior dorsal margin of first chaetiger papillated, as following segments; no other modification. Anterior chaetigers without longer papillae, chaetiger 1 shorter than following ones, chaetal lobes lateral, very close to each other. Chaetigers 5–10 swollen, without marked segmentation between them; therafter segments better defined. Gonopores orange-red, in chaetigers 2–12 (Fig. 9C, D).

Parapodia poorly developed; chaetae emerge from the body wall (Fig. 9F). Notopodia and neuropodia with papillae as long as the others. Noto- and neuropodia close to each other. Notochaetae multiarticulated capillaries, all articles long (Fig. 9G). Median notochaetae arranged in a longitudinal line, with 4 per bundle in holotype (11 per bundle in larger; 6–7 in smaller specimens), longest about as long as one-third body width. Median neuropodia lateral, very close to notopodia. Neurochaetae multiarticulated hooks from chaetiger 1 (Fig. 9G), arranged in a short longitudinal line (J-pattern in other specimens), 3–4 per bundle (6–8 in other specimens), each with long articles of about the same length, distal article falcate, finely transversely divided, not articulated, with a hood-like membrane.

Posterior end missing in holotype (probably invaginated); a posterior fragment (ZISP-48507, Fig. 9E) tapering to a rounded lobe; pygidium with anus dorsoterminal, dark, muscular, without anal cirri.

#### Variation.

Living specimens dark-orange, gills green. The paratypes were orange-yellow to orange-brown, with 29–31 chaetigers.

#### Remarks.

*Diplocirrus nicolaji* (Buzhinskaja, 1994), comb.n. is closely allied to *Diplocirrus* *branchiatus* (Rullier, 1965) because the bodies of these species lack sediment particles, have ventrolateral gonopores in some anterior chaetigers, reduced chaetae in the first chaetiger, and their caruncles taper posteriorly. Their main differences rely on the relative neurochaetal development in median chaetigers, and on the area covered by lamellae in the cirriform branchiae; thus, *Diplocirrus nicolaji* has barely tapering neurochaetae, with some 10 articles of about the same length, tips markedly falcate, and their cirriform branchiae has a lamellate region extending up to one-third of the branchial length, whereas in *Diplocirrus branchiatus*, the neurochaetae are tapering, provided with about 23 articles, decreasing in size distally, tips delicately falcate, and the lamellate region along cirriform branchiae might reach one-fifth of the branchial length.

#### Distribution.

Originally described from Vostok Bay, Peter the Great Bays, Northwestern Sea of Japan, in shallow water soft bottoms (3–7 m).

### Diplocirrus normani

(McIntosh, 1908) comb. n.

http://species-id.net/wiki/Diplocirrus_normani

Fig. 10

Stylarioides normani McIntosh 1908:542–543, Pl. 12, Figs. 3, 8.
Stylarioides longisetosus von Marenzeller 1892:426–427 (*non* von Marenzeller, 1890).
Diplocirrus longisetosus : Haase 1915:200–202, Textfigs. 6–7 (*partim*); Støp-Bowitz 1948a:30–33, Fig. 8; Støp-Bowitz 1948b:38–39, map (*non* von Marenzeller, 1890).

#### Type material.

**Barents Sea.** Holotype of *Stylarioides normani* (BMNH-1921.5.1.2646), Finmark, Northern Norway, Stat. 49, 1890, C. Norman, coll. (anterior fragment, dried-out, 7.5 mm long, 1.5 mm wide, cephalic cage 3 mm long, 14 chaetigers; right chaetiger 10 previously removed).

#### Additional material.

**Barents Sea.** Two specimens (ECOSUR), White Sea, Russia, 5 Aug. 1999, A. Filippova, coll. (complete specimen used for redescription; anterior fragment 6 mm long, 1.5 mm wide, cephalic cage 2 mm long, 14 chaetigers). One specimen (ECOSUR), White Sea, Russia, 15 m, Jul. 1999, A. Filippova, coll. (anterior fragment 3 mm long, 1 mm wide, cephalic cage 2.3 mm long, 11 chaetigers). Two specimens (ECOSUR) complete, slightly damaged, Kandalalsha Bay, White Sea, Russia, 40 m, mud, 1 Aug. 2004, A. Zhadan, coll. (specimen with anterior end exposed used for description; 5.0–5.5 mm long, 0.8–1.0 mm wide, cephalic cage 1.0–1.3 mm long, 20–21 chaetigers; exposed anterior end 0.7 mm long). **Northwestern Atlantic Ocean.** Several specimens (USNM-48491), Cape Cod Bay, Massachusetts, Stat. 1424, 35.1–33.6 m, 19 Nov. 1968, C.D. Long. Coll. Id.

#### Description.

**Figure 10.**
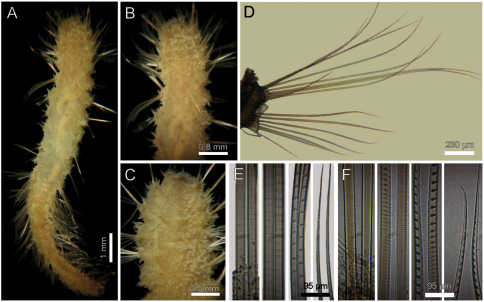
*Diplocirrus normani* (McIntosh, 1908), comb. n., reinst. Non-type specimen (ECOSUR): **A** complete, dorsal view **B** same, anterior end, dorsal view **C** same, anterior end, ventral view **D** same, chaetiger 13, right parapodium **E** same, basal to distal notochaetal regions **F** same, basal to distal neurochaetal regions.

Non-type specimen (ECOSUR) complete, yellowish gray. Body club-shaped, anteriorly swollen, slightly narrowing to chaetiger 13, then apparently regenerating the posterior, cylindrical region, tapering to posterior end (Fig. 10A); 12 mm long, 1.5 mm wide, cephalic cage 3 mm long, 25 chaetigers. Tunic papillated, with fine sediment particles. Papillae eroded, core and tips black, cirriform, sparse, fragile, about 7–8 transverse rows in median chaetigers, becoming longer dorsally (Fig. 10B); in median chaetigers about 1/5–1/6 as long as notochaetae.

Cephalic tube long, smooth, margin apparently smooth. Prostomium low, eyes not seen. Caruncle not seen. One palp remaining, thick, longer than remaining branchiae, longitudinal furrow shallow; palp keels reduced. Dorsal and ventral lips reduced, lateral lips thicker. Branchiae cirriform, most lost, sessile on branchial plate, arranged in two concentric rows, distal row continuous with 4 thicker filaments bases, proximal row discontinuous, filaments probably thinner, lower filaments bases smaller. Nephridial lobes very thin, long, placed below the posterior row lateral filaments.

Cephalic cage chaetae ¼ as long as body length, or twice as long as body width. Chaetigers 1–2 involved in the cephalic cage; chaetae arranged in short dorsolateral lines, 6–8 noto- and 4–6 neurochaetae per bundle. Anterior dorsal margin of first chaetiger papillated, black; anterior chaetigers without especially long papillae. Chaetigers 1–3 of about the same length. Post-cephalic cage chaetigers not elongated. No chaetal transition from cephalic cage to body chaetae, all neurochaetae similar. Gonopodial lobes present in chaetiger 5, low, rounded, black, covered by small papillae (Fig. 10C).

Parapodia lateral, poorly developed, chaetae emerge from the body wall (Fig. 10D); median neuropodia ventrolateral. Notopodia 2–3 with very low conical lobes directed forward, remaining notopodia less prominent. Neuropodia 2–5 with low conical chaetal lobes. Noto- and neuropodia distant to each other.

Median notochaetae arranged in a transverse horizontal C-shaped pattern; all notochaetae multiarticulated capillaries, short articles basally and distally, long medially (Fig. 10E). About 10 (–12) chaetae per bundle, at least twice as long as body width. All neurochaetae multiarticulated capillaries, short, poorly-defined articles along basal half or 2/3 chaetal length, better-defined, medium-sized and then long articles along the rest of chaetae (Fig. 10F), tips straight, arranged in a transverse line, 9–10 per bundle.

Posterior end tapering to a rounded lobe; pygidium with anus terminal, blackish, without anal cirri.

#### Remarks.

*Diplocirrus normani* (McIntosh, 1908), comb. n. was regarded as a junior synonym of *Diplocirrus longisetosus* (von Marenzeller, 1890) by Haase (1915:200) because they are very similar. As stated above, they also resemble *Diplocirrus micans* Fauchald, 1972, though the latter separates from the other two species because it lacks gonopodial lobes and its neurochaetae have long articles. Thus, once *Diplocirrus longisetosus* has been restricted, these species differ regarding coloration of body, papillae and gonopodial lobes, and because of the relative resolution of neurochaetal basal articles. Thus, in *Diplocirrus normani*, although the body is grayish, papillae and gonopodial lobes are darker or blackish, and neurochaetal basal articles are poorly-defined, whereas in *Diplocirrus longisetosus*, on the contrary, the papillae and gonopodial lobes are pale, and the basal articles of neurochaetae are well-defined.

#### Distribution.

Originally described from Finmark, Northern Norway, Barents Sea. It ranges along Northeastern and Northwestern Atlantic areas, in shallow water.

### Diplocirrus octobranchus

(Hartman, 1965) comb. n.

http://species-id.net/wiki/Diplocirrus_octobranchus

Fig. 11

Ilyphagus octobranchus Hartman 1965:178–179, Pl. 39; Hartman and Fauchald 1971:120–121.
Diplocirrus octobranchus :Day 1973:107 (informal comb. n.); Darbyshire and Mackie 2009:97, Table 1.

#### Type material.

**Eastern Atlantic Ocean.** Holotype (LACM-AHF 540) and 19 paratypes (LACM-AHF 541), off New England, United States, RV Atlantis Stat. Slope 3 (39°58'24"N, 70°41'18"W), 300 m, 28 Aug. 1962, H. Sanders, coll. (two complete paratypes 7–16 mm long, 0.8–1.0 mm wide, cephalic cage 0.8–2.0 mm long, 24–42 chaetigers; gonopodial papillae not visible; smaller paratypes with relatively more sand particles over their bodies; broken mature female with oocytes about 120 µm).

#### Additional material.

**North Carolina.** One specimen (USNM-54938), Eastward Stat. 6269 (34°16.5'N, 75°44'W), 500–520 m, 11 Nov. 1966, G. Rowe, coll. One specimen (USNM-54932), Eastward Stat. 6241 (33°13.6’ N, 76°13.4’ W), small biological trawl, 1000–1020 m, 9 Nov. 1966, G. Rowe coll.

#### Description.

**Figure 11.**
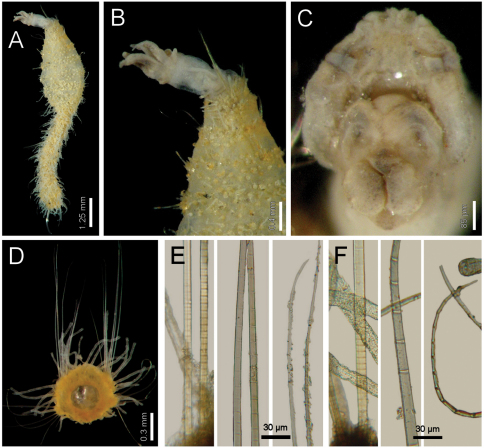
*Diplocirrus octobranchus* (Hartman, 1965), comb. n. **A** holotype (LACM-AHF 540), dorsal view **B** same, anterior end, dorsal view **C** paratype (LACM-AHF 541), head, frontal view, palps and branchiae removed **D** same, chaetiger 18 **E** same, basal, medial and distal notochaetal regions **F** same, basal, medial and distal neurochaetal regions.

Holotype an anterior fragment, brownish. Body anteriorly swollen, posteriorly tapered (Fig. 11A); 8.5 mm long, 1 mm wide (widest by chaetigers 5–6, 2 mm), cephalic cage 2 mm long, 17 chaetigers. Tunic papillated, sediment particles mostly fine, adherent on papillae bases, and few larger sand grains, especially dorsally (Fig. 11B); smaller specimens with more sand particles on the body. Papillae of varying lengths, longer dorsally and on chaetal lobes, may be as long as chaetae, shorter in the rest of the body, 4–5 rows per chaetiger.

Cephalic hood tube long, made of two rings, basal one shorter, both smooth; cephalic hood margin smooth. Prostomium low, eyes not seen (Fig. 11C). Caruncle low, wide. Palps lost (pale, laterally corrugated, 1.5 times longer than branchiae in one paratype); palp keels rounded, elevated. Lateral lips thick, projected outwards, rounded. Ventral lip reduced. Dorsal lip projected as a triangular lobe. Branchiae cirriform of two different widths; posterior row with thicker filaments, rectangular, with a middorsal black band, branchial bases continuous, anterior row with branchiae thinner, cirriform, separated in two lateral pairs. Branchiae of about the same length; size relationships with palps unknown. Nephridial lobes in branchial plate low, whitish.

Cephalic cage chaetae as long as widest body section. Only chaetiger 1 involved in the cephalic cage; chaetae arranged in a short, dorsolateral line with 4(–5) noto- and 2(–8 in paratypes) neurochaetae. Anterior dorsal margin of first chaetiger papillated. Chaetigers 1–3 progressively larger. Post-cephalic cage chaetigers not elongated. Chaetal transition from cephalic cage to body chaetae abrupt, thicker neurospines present from chaetiger 2. Gonopodial lobes not seen.

Parapodia poorly-developed, chaetae emerge from the body wall (Fig. 11D). Parapodia lateral; median neuropodia ventrolateral. Noto- and neuropodia without projected chaetal lobes. Papillae abundant, 2–4 larger ones in chaetal lobes. Noto- and neuropodia close to each other.

Median notochaetae arranged in a short transverse line, chaetae directed dorsally. All notochaetae multiarticulated capillaries. Median notochaetae 1.5–2.0 times as long as body width, 7 per bundle, articles short basally, feebly defined, become medium-sized medially, long distally (Fig. 11E). Neurochaetae multiarticulated capillaries in chaetiger 1; thicker multiarticulated neurospines from chaetiger 2, two (–5 in paratypes) per ramus, become thinner in the tapered median and posterior region, being 5 per ramus, arranged in a transverse line. Neurochaetae with feebly-defined short, basal articles, become very long medially, and decrease progressively to the straight tip (Fig. 11F).

Posterior end observed in one complete paratype, tapering to a swollen pygidium, with anus dorsoterminal, without anal cirri. One paratype is a damaged female, oocytes 100–150 µm.

#### Remarks.

*Diplocirrus octobranchus* (Hartman, 1965), comb. n., is closely allied to an undescribed species from Antarctica, and both differ from other species with long papillae because they have sand particles over the body. These two species differ in the extent of sediment particles along the papillae and on the relative length of the neurochaetal anchylosed region. Thus, in *Diplocirrus octobranchus* sediment particles are restricted to the base of papillae, and their neurochaetae have an anchylosed region of about one-fifth of the chaetal length, whereas in the Antarctic undescribed species, the sediment particles spread along the papillae, and the anchylosed region might be about half or one-third of the chaetal length.

*Diplocirrus octobranchus* is a typical member of the genus because its branchiae are of two different widths. It does not belong in *Ilyphagus* because it has multiarticulated neurospines, with long articles in the medial and distal regions, and short articles only basally, whereas in *Ilyphagus* neurochaetae are aristate spines with very short articles basal- and medially, and distally hyaline. Further, the cephalic cage chaetae in *Ilyphagus* are clearly dorsal whereas in *Diplocirrus* they are lateral, or dorsolateral at most. After Hartman amended *Ilyphagus* (Hartman 1965:177), the correct placement for her new species as a member of *Diplocirrus* was indirectly stated by comparing it to *Diplocirrus glaucus*, the type species for the genus (Hartman 1965:179). This made Day (1973:106) suggest the informal, new combination, which is herein confirmed after the examination of the type material and of the redefinition of *Diplocirrus*.

#### Distribution.

Apparently discontinuous; off New England in 300–1000 m, and off northeastern South America in 770–805 m.

### Diplocirrus stopbowitzi

Darbyshire & Mackie, 2009

http://species-id.net/wiki/Diplocirrus_stopbowitzi

Fig. 12

Diplocirrus stopbowitzi Darbyshire and Mackie 2009:93–96, Figs. 1–3A, Table 1.

#### Material examined.

One specimen, broken in three pieces, Stat. BSA 449, 12 mm long, 0.8 mm wide, 25 chaetigers, A. Ravara, coll. (no further data available).

#### Diagnosis.

**Figure 12.**
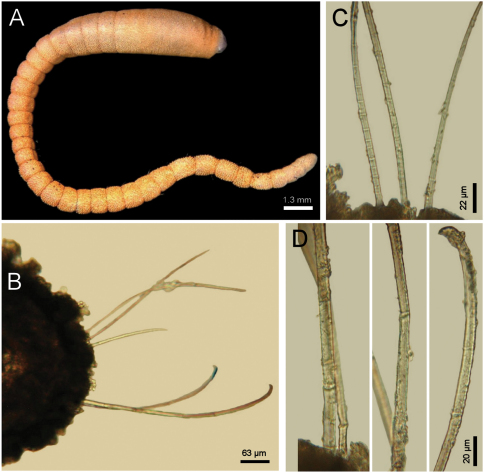
*Diplocirrus stopbowitzi* Darbyshire & Mackie, 2009. Non-type specimens **A** complete, lateral view (photo by Teresa Darbyshire) **B** another specimen, chaetiger 20, left parapodium **C** same, notochaetal basal regions **D** same, basal, medial and distal neurochaetal regions.

Body slightly swollen anteriorly (Fig. 12A). Papillae abundant, short, giving a velvety oultlook, without sediment particles (Fig. 12B). Median chaetigers with 5–6 notochaetae and 2–3 neurochaetae; posterior chaetigers with three notochaetae and two neurochaetae. Notochaetae with long articles throughout the chaeta (Fig. 12C). Neurochaetae with long articles, being 7–8 times longer than wide, tips falcate (Fig. 12D).

#### Remarks.

As stated above, *Diplocirrus stopbowitzi* Darbyshire & Mackie, 2009 resembles *Diplocirrus* *kudenovi* sp. n. because in both species the body has hemispherical papillae, but lacks sand particles or ventrolateral gonopores. They especially differ regarding some neurochaetal features in median chaetigers such as their number and the relative length of articles; thus, *Diplocirrus stopbowitzi* has 2–3 neurochaetae, each with long articles being about seven times longer than wide, whereas *Diplocirrus kudenovi* has 5–6 neurochaetae and each has shorter articles, each being twice as long as wide.

#### Distribution.

Southern Irish Sea, offshore, in gravel or gravelly-sand bottoms, 38–112 m depth.

### Diplocirrus sp. n. Antarctica

#### Material examined.

One specimen (USNM 46405), without posterior region, RV Staten Islands, Stat. 9-63 (64°48'S, 63°30'W), Port Lockroy, off Wiencke Island, Anvers Island, 31 fathoms, dredged at anchorage, mud bottom, 26 Jun. 1963, W.L. Schmitt, coll. (5.5 mm long, 0.7 mm wide, cephalic cage 0.9 mm long, 20 chaetigers). One slide with three segments (USNM 56470).

#### Remarks.

This undescribed species is closely allied to *Diplocirrus octobranchus* because both have sediment particles on the body and 7–8 notochaetae per bundle in median chaetigers. They differ because in *Diplocirrus* sp Antarctica, the sediment particles are adhered in the body wall and in the whole papillae, whereas in *Diplocirrus octobranchus*, sediment particles are restricted to the base of the papillae leaving bare both the body wall and the papillae. Another important difference is the extension of the anchylosed articles; thus, the anchylosed portion is one-half or at least one-third of notochaetal length in the Antarctic species, whereas it is only one-fifth or less of neurochaetal length in *Diplocirrus octobranchus*.

### Diplocirrus sp. n. Morocco

*Stylarioides scutigeroides*: Fauvel 1936:77 (*partim*, *non* Augener, 1918).

#### Material examined.

**Morocco.** Two anterior fragments (MNHN-361), most chaetae broken, RV Vanneau, Stat. 6 (31°42'N, 09°43'W), 22 m, 1 Jul. 1923, R.P. Dollfus & J. Liouville, coll. (5.0–5.5 mm long, 1.5 mm wide, cephalic cage 1.5 mm long, 12 chaetigers; anterior end dissected, it has the typical *Diplocirrus* pattern; i.e. 8 branchial filaments with the posterior ones thicker).

#### Remarks.

This species differs from other species with short papillae because *Diplocirrus* sp. Moroccohas very short lateral papillae and the body wall has a thin layer of sediment grains. However, there are no more specimens available from the same expedition.

#### Distribution.

Only known from off Cape Guir, Morocco, in 22 m depth.

### Diplocirrus sp. n. Sri Lanka

#### Material examined.

Three specimens (MNHN-unnumb.), off SW Sri-Lanka, RV Marion Dufresne, SAFARI II Cruise, Stat. 2 (05°37'N, 78°24'E), 3660 m, Jul. 1981.

#### Description.

Three anterior fragments variously damaged. Body cylindrical, tapering posteriorly; 2.5–3.5 mm long, 0.7–1.2 mm wide, cephalic cage (broken) 1 mm long, 8–10 chaetigers. Tunic thin, without foreign particles, with 4 longitudinal rows of elongate papillae.

Cephalic hood not exposed. Anterior end not dissected to avoid further damage. Cephalic cage chaetae about as long as body width. Chaetiger 1 involved in the cephalic cage; chaetae in short ventrolateral lines, 1–2 noto- and 2–3 neurochaetae per ramus. Anterior dorsal margin of first chaetiger papillated, papillae elongate, clavate. Anterior chaetigers without especially long papillae. Chaetigers 1–3 of about the same length. Chaetal transition from cephalic cage to body chaetae abrupt; thicker neurochaetae start in chaetiger 2. Gonopodial lobes not seen.

Parapodia poorly-developed, chaetae emerge from the body wall. Parapodia lateral; median neuropodia ventrolateral. Noto- and neuropodia close to each other, each with 2–3 longer clavate papillae. Median notochaetae arranged in a tuft, most broken; all notochaetae multiarticulated capillaries, articles long; in median chaetigers 2–3 per bundle, as long as 2/3 body width. Neurochaetae multiarticulated capillaries in chaetiger 1; thicker articulated neurospines from chaetiger 2, with articles short basally, medial- and distally long, 2–3 per bundle.

Posterior end unknown.

#### Remarks.

With the available specimens and as indicated in the key above, this species differs from all other species in the genus because it has a rather smooth body. Better specimens would clarify its affinities and allow a description.

#### Distribution.

Only known from the type locality, off Sri-Lanka, in 3660 m depth.

## Supplementary Material

XML Treatment for Diplocirrus

XML Treatment for Diplocirrus glaucus

XML Treatment for Diplocirrus branchiatus

XML Treatment for Diplocirrus capensis

XML Treatment for Diplocirrus erythroporus

XML Treatment for Diplocirrus hirsutus

XML Treatment for Diplocirrus incognitus

XML Treatment for Diplocirrus kudenovi

XML Treatment for Diplocirrus longisetosus

XML Treatment for Diplocirrus micans

XML Treatment for Diplocirrus nicolaji

XML Treatment for Diplocirrus normani

XML Treatment for Diplocirrus octobranchus

XML Treatment for Diplocirrus stopbowitzi
